# Secondary Metabolites from Marine-Derived Fungus *Penicillium rubens* BTBU20213035

**DOI:** 10.3390/jof10060424

**Published:** 2024-06-16

**Authors:** Xiuli Xu, Yifei Dong, Jinpeng Yang, Long Wang, Linlin Ma, Fuhang Song, Xiaoli Ma

**Affiliations:** 1 Key Laboratory of Marine Mineral Resources and Polar Geology, Ministry of Education, School of Ocean Sciences, China University of Geosciences, Beijing 100083, China; 2 Key Laboratory of Geriatric Nutrition and Health, Ministry of Education of China, School of Light Industry Science and Engineering, Beijing Technology and Business University, Beijing 100048, China; 3 State Key Laboratory of Mycology, Institute of Microbiology, Chinese Academy of Sciences, Beijing 100101, China; 4 Griffith Institute for Drug Discovery, School of Environment and Science, Griffith University, Brisbane 4111, Australia; 5 School of Chemistry and Chemical Engineering, North Minzu University, Yinchuan 750021, China

**Keywords:** marine-derived fungus, *Penicillium rubens*, polyketide, bis-tetrahydrofuran, antibacterial, synergistic antifungal

## Abstract

Two new polyketide derivatives, penirubenones A and B (**1** and **2**), and two naturally rare amino-bis-tetrahydrofuran derivatives, penirubenamides A and B (**3** and **4**), together with nine known compounds (**5**–**13**) were isolated from the marine-derived fungus *Penicillium rubens* BTBU20213035. The structures were identified by HRESIMS and 1D and 2D NMR analyses, and their absolute configurations were determined by a comparison of experimental and calculated electronic circular dichroism (ECD) spectroscopy and ^13^C NMR data. We found that **6** exhibited antibacterial activity against *Staphylococcus aureus*, with an MIC value of 3.125 μg/mL, and **1** and **2** showed synergistic antifungal activity against *Candida albicans* at 12.5 and 50 μg/mL with 0.0625 μg/mL rapamycin.

## 1. Introduction

Over the past several decades, pathogenic fungi have had a devastating impact on human health. As invasive pathogens, *Candida* species rank as a leading cause of healthcare-associated bloodstream infections in the United States [1,2,3]. Infective diseases caused by *Candida* species result in a high mortality rate (40–60%), leading to an estimated 400,000 deaths globally per year [4]. There are five classes of antifungal drugs currently available for treating fungal infections [5]. However, with the worldwide spread of drug-resistant strains, pathogenic fungi kill about 1.5 million individuals annually [6,7]. Thus, the development of novel antifungal drugs has become an urgent requirement for researchers and clinicians [8]. To overcome the challenge of drug resistance in pathogenic microbes, the combination of two compounds, or synergism, has attracted increasing research interest [9,10].

Marine natural products, known for their high chemical biodiversity and potent bioactivity, have served as valuable new resources for the development of antifungal drugs [11,12,13]. Nowadays, natural products isolated from marine fungi are predominant in marine natural products, with over 30% of new compounds identified from marine-derived fungi [14,15]. *Penicillium*, famed for the discovery of the first antibiotic, penicillin, continues to be an attractive microbial source for new chemical entries. New compounds with antimicrobial, anti-inflammatory, cytotoxic, and other bioactivities with potential applications in drug development have been reported [16,17,18]. 

In the course of our continuous investigation of the secondary metabolites of marine-derived fungi [19,20,21], four new compounds named penirubenones A and B (**1** and **2**) and penirubenamides A and B (**3** and **4**), along with the nine known compounds penimeroterpenoid A (**5**), 2,4′-linked secalonic acid (**6**), dihydrocitreohybridonol (**7**), 3-deacetylated andrastin A (**8**), citreohybridonol (**9**), penicyclone A (**10**), penicyclone D (**11**), penicyclone E (**12**), and peniginsengin A (**13**), were isolated from a marine-derived fungus strain of *Penicillium rubens* BTVU20213035. The in vitro antimicrobial and synergistic antifungal activities of the isolated compounds were evaluated against *Candida albicans*, *Staphylococcus aureus*, and *Escherichia coli*, respectively. Herein, we report in detail the performance of isolation, structure elucidation, and bioactivity assays for these compounds.

## 2. Materials and Methods

### 2.1. Molecular Identification

The strain BTBU20213035 was isolated from a mud sample collected from the intertidal zones of Sanya, Hainan Province, China. The fungus strain was inoculated on a malt extract agar plate and cultured at 28 °C for 7 days, resulting in colonies about 10 mm in diameter. The genomic DNA of BTBU20213035 was extracted using a DNA quick Plant System (Tiangen), and the ITS sequence was amplified using the conventional primer pair of ITS4 (5′-TCCTCCGCTTATTGATATGC-3′) and ITS5 (5′-GGAAGTAAAAGTCGTAACAAGG-3′). PCR products were sequenced by Beijing Qingke Biotechnology Co., Ltd. (Beijing, China). The strain BTBU20213035 was identified by comparing ITS sequences with data from the GenBank database using the BLAST program. Alignments and calculations of sequence similarity were carried out using CLUSTAL W [22]. The strain was deposited at Beijing Technology and Business University, Beijing, China, with the accession number BTBU20213035.

### 2.2. General Experimental Procedure

HR-ESI MS was measured by an Accurate-Mass-Q-TOF LC/MS 6520 instrument (Santa Clara, CA, USA). NMR spectra were recorded on a Bruker Avance 500 spectrometer (Bruker, Germany) with residual solvent peaks as references. Optical rotations were measured by using an Anton Paar MCP 200 Modular Circular polarimeter (Austria) in a 100 × 2 mm cell at 25 °C. Thin-layer chromatography silica (Qingdao Haiyang Chemical Co. Ltd., Qingdao, China) and Sephadex LH-20 (GE Healthcare, Uppsala, Sweden) were used for column chromatography. Semipreparative HPLC was performed on an Agilent 1200 HPLC system equipped with an Agilent DAD UV-vis spectrometric detector, using a reversed-phase column (C8, 5 μm, 9.4 mm × 250 mm, Agilent, Santa Clara, CA, USA).

### 2.3. Fungal Materials, Cultivation, Fermentation, and Isolation

*P. rubens* BTBU20213035 was inoculated on a malt extract agar plate and incubated at 28 °C for 7 days. A slice of fungal colony (1 cm^2^) was cut from the plate and put into three conical flasks (250 mL each) containing 50 mL yeast peptone dextrose medium and cultured at 28 °C (180 rpm) for 36 h. Then, 5 mL of the cultured medium was inoculated into fifteen 1 L conical flasks, each containing a sterilized medium of 120 g rice and 120 mL distilled water. The inoculated flasks were incubated in a stationary manner at 28 °C for 28 days. The fermented materials were extracted three times by EtOAc:MeOH (80:20), and the organic solvent was evaporated in vacuo at 45 °C to yield a brown crude extract. The crude extract was resuspended in 500 mL distilled water and extracted with 500 mL EtOAc (three times). Then, EtOAc was evaporated in vacuo at 45 °C to give a dark residue (17.16 g). The EtOAc extract was separated by vacuum liquid chromatography (50 × 80 mm column, silica gel H for thin-layer chromatography) with a stepwise gradient of 50–100% hexane/CH_2_Cl_2_ and then 99–0% CH_2_Cl_2_/MeOH to produce 13 fractions. The masses of the fractions were 1136.0 mg, 3847.8 mg, 1400.2 mg, 1090.8 mg, 471.5 mg, 298.4 mg, 432.6 mg, 305.3 mg, 1248.3 mg, 1182.4 mg, 1138.3 mg, 739.0 mg, and 322.7 mg, respectively. The ninth fraction was subjected to a Sephadex LH-20 column using an elution of CH_2_Cl_2_:MeOH (2:1) to give nine subfractions. The fifth subfraction was further separated by HPLC (Agilent Eclipse XDB-C8, 250 × 9.4 mm, 5 μm column, 3.0 mL/min) eluted with 40–70% MeCN/H_2_O for 25 min, then with 100% MeCN for 35 min, to yield compounds **1** (*t*_R_ 9.73 min, 2.7 mg), **5** (*t*_R_ 19.42 min, 5.6 mg) and **6** (*t*_R_ 22.46 min 15.5 mg). The tenth fraction was purified on a Sephadex LH-20 column using an elution of CH_2_Cl_2_:MeOH (2:1) to give nine subfractions. The fifth subfraction was further separated by HPLC (Agilent Eclipse XDB-C8, 250 × 9.4 mm, 5 μm column, 3.0 mL/min) eluted with 30% MeCN/H_2_O for 8 min, then with 50% MeCN/H_2_O for 25 min, then with 100% MeCN for 40 min to yield compounds **7** (*t*_R_ 11.34 min, 3.0 mg), **8** (*t*_R_ 13.52 min, 2.1 mg), **9**, **3** (*t*_R_ 15.00 min, 3.3 mg), and **4** (*t*_R_ 28.53 mi, 2.6 mg), (*t*_R_ 28.960,15.5 mg). The sixth subfraction was further separated by HPLC (Agilent Eclipse XDB-C8, 250 × 9.4 mm, 5 μm column, 3.0 mL/min) eluted with 30% MeCN/H_2_O for 8 min, then with 50% MeCN/H_2_O for 25 min, then with 100% MeCN for 40 min to yield compounds **10** (*t*_R_ 5.02 min, 11.6 mg), **2** (*t*_R_ 7.89 min, 2.1 mg), **11** (*t*_R_ 8.23, 5.9 mg), **12** (*t*_R_ 19.97, 4.2 mg), and **13** (*t*_R_ 24.99, min 3.6 mg).

Penirubenone A (**1**): Yellow powder; ${\left[a\right]}_{25}^{D}$ (*c* 0.1, +19.0, MeOH); ^1^H and ^13^C NMR data (Table 1); HRESIMS *m*/*z* 355.1522 [M + Na]^+^ (calcd for C_19_H_24_O_5_Na^+^, 355.1516).

Penirubenone B (**2**): Pink powder; ${\left[a\right]}_{25}^{D}$ (*c* 0.1, −15.0, CHCl_3_); ^1^H and ^13^C NMR data (Table 1); HRESIMS *m*/*z* 289.1052 [M + Na]^+^ (calcd for C_14_H_18_O_5_Na^+^, 289.1046).

Penirubenamide A (**3**): ${\left[a\right]}_{25}^{D}$ (*c* 0.1, −13.0, MeOH); ^1^H and ^13^C NMR data (Table 2); HRESIMS *m*/*z* 380.2435 [M + H]^+^ (calcd for C_19_H_24_O_5_Na^+^, 380.2431).

Penirubenamide B (**4**): ${\left[a\right]}_{25}^{D}$ (*c* 0.1, −18.0, MeOH); ^1^H and ^13^C NMR data (Table 2); HRESIMS *m*/*z* 366.2640 [M + H]^+^ (calcd for C_19_H_24_O_5_Na^+^, 366.2639).

### 2.4. ECD Calculation Methods

A random conformation search of starting geometries in GaussView 6 was used to produce low-energy conformers within 5 kcal/mol of energy, through which 6, 4, and 4 conformers were obtained for **1**-**1, 1**-**2**, and **2**, respectively. The ECD calculation was performed with the software package Gaussian 16 using the DFT method at the B3LYP/6-31G(d) level [23]. TDDFT (time-dependent density functional theory) calculations of their low-energy conformations were performed using a solvent model in methanol to simulate their UV–vis spectra at the same level. Origin 9.0 was used to compare the calculated and experimental CD curves. ^13^C NMR calculations were carried out via the GIAO method at the same level [24]. Statistical parameters, including correlation coefficient (*R*^2^), mean absolute error (MAE), and maximum error (MaxErr), were used to quantify the agreement between the experimental and calculated data [25]. The correlation coefficient (*R*^2^) was determined from a plot of *δ*_calc_ (*x* axis) against *δ*_exp_ (*y* axis) for each particular compound.

### 2.5. Antibacterial Assay

The antibacterial activities against *Escherichia coli* and *Staphylococcus aureus* were determined in a 96-well plate using the microdilution method in LB broth medium. The compounds and the positive control were dissolved in dimethyl sulfoxide (DMSO) with nal concentrations ranging from 0.39 to 200 µg/mL using a 2-fold serial dilution method. The test strains were incubated with the diluted compounds at 37 °C for 18 h. DMSO, ciprofloxacin, and vancomycin (ranging from 0.0625 to 4 µg/mL) served as negative and positive controls, respectively.

### 2.6. Antifungal Assay

The assessment of antifungal activity against *C. albicans* was carried out in 96-well plate format using the microdilution method in RPMI 1640 medium [26]. The compounds were dissolved in dimethyl sulfoxide (DMSO) at final concentrations that ranged from 0.39 to 200 µg/mL by using a 2-fold serial dilution method. The test strains were incubated with the diluted compounds at 28 °C for 18 h. DMSO and rapamycin (ranging from 0.125 to 4 µg/mL) were used as negative and positive controls, respectively.

### 2.7. Synergistic Antifungal Assay

Synergistic antifungal activity was determined by combining a positive control, rapamycin, with the isolated compounds at different concentrations. The compounds and rapamycin were dissolved in DMSO. The concentrations of the isolated compounds and rapamycin ranged from 0.39 to 200 μg/mL and from 0.03125 to 4 μg/mL, respectively. In the 96-well plate, the tested compounds were diluted 2-fold across columns 1 to 10, while rapamycin was diluted 2-fold across rows A to G. The fractional inhibitory concentration index (FICI) was defined as the sum of the minimal inhibitory concentration (MIC) of each tested sample used in combination divided by the MIC of the drug used alone. Synergistic activity was defined as an FICI ≤ 0.5 [27]. The MIC was defined as the minimum concentration of compound at which no bacterial growth was observed.

## 3. Results and Discussion

### 3.1. Phylogenetic Analysis

**Figure 1:**
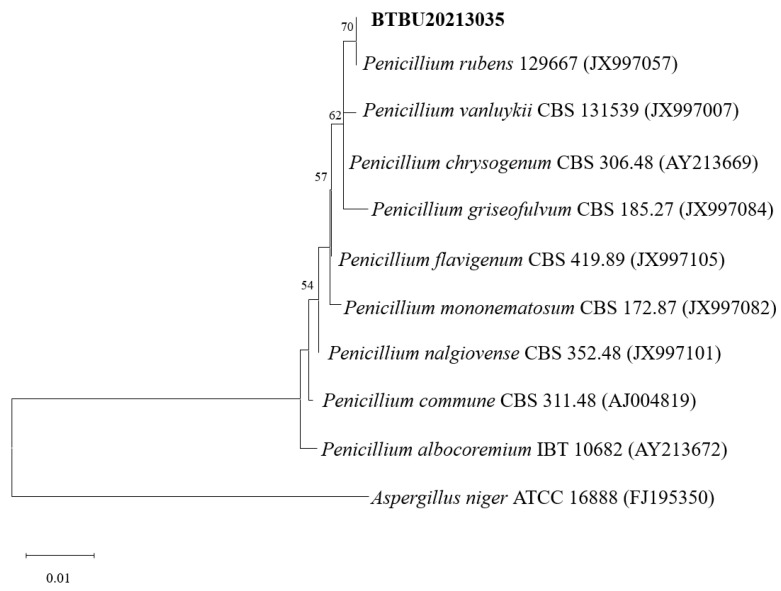
Maximum likelihood analysis based on ITS sequences. Bootstrap values ≥ 75% are indicated at the nodes. The tree was rooted to *A. Niger* ATCC 16888.

A phylogenetic tree based on ITS rDNA sequences was constructed and is shown in Figure 1. The strain BTBU202130355 shared the highest identity (99.64%) with *P. rubens* CBS 129667. Phylogenetically, the strain BTBU20213035 was identified as belonging to the known strain of *P. rubens*.

### 3.2. Structure Elucidation

**Figure 2:**
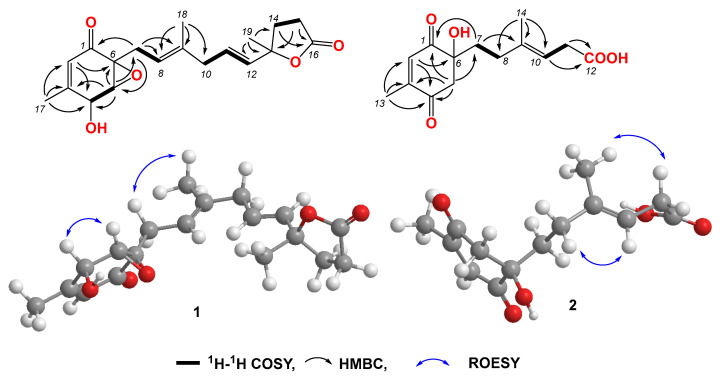
Key COSY, HMBC, and ROESY correlations in compounds **1** and **2**.

**Figure 3:**
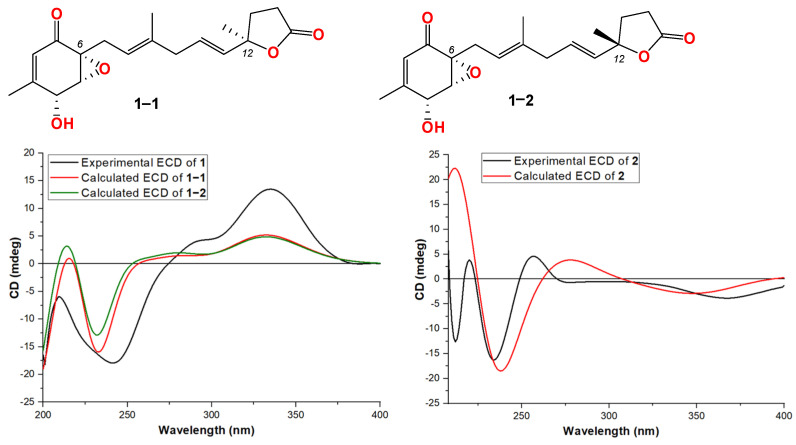
Structures and experimental ECD spectra of **1** and **2**.

Compound **1** was obtained as a light-yellow powder. The molecular formula of compound **1** was determined to be C_19_H_24_O_5_ based on its high-resolution electrospray ionization mass spectrum (HRESIMS) (*m*/*z* [M+Na]^+^ 355.1522, calcd. for C_19_H_24_O_5_^+^, 355.1516), accounting for five degrees of unsaturation (Figure S1). The ^1^H NMR spectrum (Figure S2, Table 1) of compound **1** showed signals for four olefinic protons at *δ*_H_ 5.72 (1H, t, *J* = 1.5 Hz), 5.65 (1H, overlap), 5.62 (1H, overlap), and 5.11 (1H, m); two oxygenated methines at *δ*_H_ 4.51 (1H, m) and 3.64 (1H, d, *J* = 3.0 Hz); four methylene groups at *δ*_H_ 2.74 (2H, overlap), 2.74 (1H, overlap), 2.44, dd, *J* = 15.0, 7.0 Hz), 2.57 (2H, m), 2.22 (1H, m), and 2.12 (1H, mb); one triplet methyl at *δ*_H_ 1.98 (3H, t, *J* = 1.0 Hz); one doublet methyl at *δ*_H_ 1.48 (3H, d, *J* = 1.5 Hz); and one singlet methyl at *δ*_H_ 1.64 (3H, brs). The ^13^C NMR and HSQC spectra (Figures S3 and S4, Table 1) of compound **1** displayed 19 carbon signals, including one unsaturated carbonyl at *δ*_C_ 195.8; one carboxyl at *δ*_C_ 179.6; four sp^2^ methines at δ_C_ 135.1, 128.9, 123.6, and 119.6; two sp^2^ quaternary carbons at δ_C_ 159.6 and 138.6; four sp^3^ methylenes at *δ*_C_ 27.4, 43.0, 34.9, and 29.8; two oxygenated methines at *δ*_C_ 68.1 and 60.7; three methyl carbons at δ_C_ 20.3, 16.5, and 26.6; and two oxygenated sp^3^ quaternary carbons at δ_C_ 87.2 and 61.7. The ^1^H-^1^H COSY correlation (Figure 2 and Figure S5) spectrum indicated the substructures of C-4/C-5, C-7/C-8, C-10/C-11/C-12, and C-14/C-15. In the HMBC spectrum (Figure S6), the correlations from H-2 (*δ*_H_ 5.72) to C-4 (*δ*_C_ 68.1), C-6 (*δ*_C_ 61.7) and C-17 (δ_C_ 20.3), and from H_3_-17 (*δ*_H_ 1.98) to C-1 (*δ*_C_ 195.8), C-2 (*δ*_C_ 123.6), C-3 (*δ*_C_ 159.6), and C-4 (*δ*_C_ 68.1), revealed the fragment of C-1/C-2/(C-3/C-17))/C-4 (Figure 2). The connection of C-8/(C-9/C-18)/C-10 was identified by HMBC signals from H_3_-18 (*δ*_H_ 1.64) to C-8 (δ_C_ 119.6), C-9 (δ_C_ 138.6), and C-10 (δ_C_ 43.0). The HMBC correlations from H_3_-19 (*δ*_H_ 1.48) to C-12 (δ_C_ 135.1), C-13 (δ_C_ 87.2), and C-14 (δ_C_ 34.9), and from H_2_-14 (δ_H_ 2.22 and 2.12) to C-16 (*δ*_C_ 179.6), C-12, C-13, C-15 (*δ*_C_ 29.8), and C-19 (*δ*_C_ 26.6), revealed the connection of C-12/(C-13/C-19)/C-14. The cyclopentenone fragment and the connection from C-6 to C-7 were confirmed by the HMBC signals from H_2_-7 (*δ*_H_ 2.74 and 2.44) to C-1, C-5 (*δ*_C_ 60.7) and C-6 (*δ*_C_ 61.7). By analyzing the chemical shifts of C-5 (60.7), C-6 (61.7), and C-13 (87.2), as well as the molecular formula, the presence of an epoxide between C-5 and C-6 and a lactone connection between C-13 and C-16 was inferred. Therefore, the planar structure of compound **1** was identified. The ROESY correlations (Figure 2 and Figure S7) between H_2_-7 and H_3_-18 revealed that C-7 and C-18 were in the same orientation as the double bond of C-8/C-9. The ROESY correlation between H-4 (*δ*_H_ 4.51) and H-5 (*δ*_H_ 3.64), together with the coupling constant of H-4 and H-5 (3.0 Hz), indicated the relative stereochemistry of C-4, C-5 and C-6 [28]. When determining the absolute configurations, C-12 provided two different relative configurations to C-6 (Figure 3). To resolve this, we compared the experimental and calculated ECD spectra (Figure 3). Both ECD spectra of **1**-**1** and **1**-**2** were similar to the experimental one, although the calculated ECD of **1**-**1** matched better than that of **1**-**2**. We then calculated the ^13^C NMR of these two possible structures using density functional theory (DFT). The chemical shifts in ^13^C NMR between the experimental and calculated data (Table 3) were analyzed using statistical parameters including the correlation coefficient (R^2^) with a linear regression, the maximum error (MaxErr), and the mean absolute error (MAE). Based on all these statistical parameters, along with the calculated ECD data, the structure of **1** was assigned as **1**-**1** and it was named penirubenone A.

Compound **2** was obtained as a pink powder. The molecular formula of compound **2** was determined to be C_14_H_18_O_5_ based on the high-resolution electrospray ionization mass spectrum (HRESIMS) (*m*/*z* [M + Na]^+^ 289.1052, calcd. for C_14_H_18_O_5_Na^+^, 289.1046), accounting for six degrees of unsaturation (Figure S8). The ^1^H NMR spectrum (Figure S9, Table 1) of compound **2** showed signals for two olefinic protons at *δ*_H_ 6.70 (1H, m) and 5.26 (1H, t, *J* = 7.0 Hz); four methylene groups at *δ*_H_ 3.17 (1H, d, *J* = 16.0 Hz), 2.92 (1H, d, *J* = 16.0 Hz), 1.87 (1H, m), 1.70 (1H, m), 2.12 (1H, m), 1.87 (1H, m), and 3.05 (2H, d, *J* = 7.0 Hz); one doublet methyl at *δ*_H_ 2.04 (3H, d, *J* = 1.5 Hz); and one triplet methyl at *δ*_H_ 1.58 (3H, t, *J* = 1.0 Hz). The ^13^C NMR and HSQC spectra (Figures S9 and S10, Table 1) of compound **2** displayed fourteen carbon signals, including two unsaturated carbonyls at *δ*_C_ 201.4 and 196.1; one carboxyl at *δ*_C_ 177.4; two sp^2^ methines at δ_C_ 134.4 and 116.2; two sp^2^ quaternary carbons at δ_C_ 152.5, 138.4 (C-9); four sp^3^ methylenes at *δ*_C_ 51.3, 38.6, 33.3, and 33.0; one oxygenated quaternary carbon at *δ*_C_ 77.9; and two methyl carbons at δ_C_ 16.6 and 16.4. These signals indicated that compound **2** contained the skeleton of penicyclone D, an ambuic acid analogue [28]. The ^1^H-^1^H COSY correlations (Figure S12 and Figure 2) revealed the substructures of C-7/C-8 and C-10/C-11. The carboxyl group of C-12 was identified by HMBC (Figure S13 and Figure 2) correlation from H_2_-11 (*δ*_H_ 3.05) to C-12 (*δ*_C_ 177.4). The connections of C-8, C-10, and C-14 to C-9 were confirmed by HMBC correlations from H_3_-14 (*δ*_H_ 1.58) to C-8 (*δ*_C_ 33.0), C-9 (*δ*_C_ 138.4), and C-10 (*δ*_C_ 116.2). The HMBC correlations from H-2 (*δ*_H_ 6.70) to C-4 (δ_C_ 196.1) and C-13 (δ_C_ 16.4), from H_3_-13 (*δ*_H_ 2.04) to C-2 (δ_C_ 134.4), C-3 (δ_C_ 152.5) and C-4, and from H_2_-5 (*δ*_H_ 3.17 and 2.92) to C-1 (*δ*_C_ 201.4), C-4, and C-6 (*δ*_C_ 77.9) revealed the substructure of methylcyclohexdione. The connection of C-6 to C-7 was established by HMBC correlations from H_2_-7 (*δ*_H_ 1.87 and 1.70) to C-1, C-5 (*δ*_C_ 51.3), and C-6. The configuration of double bond C-9/C-10 was established by ROESY correlations (Figure S14 and Figure 2) between H_2_-8 (*δ*_H_ 2.12 and 1.87) and H-10 (*δ*_H_ 5.26), and between H-11 (*δ*_H_ 3.05) and H-14 (*δ*_H_ 1.58). Therefore, the planar structure of compound **2** was determined. The calculated ECD spectrum was consistent with the experimental data (Figure 3), indicating absolute configuration of compound **2**. Therefore, the structure of compound **2** was determined and it was named penirubenone B.

**Figure 4:**
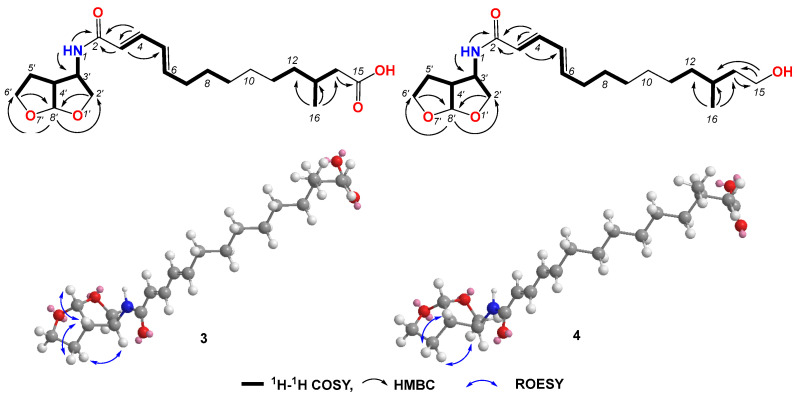
Key COSY, HMBC, and ROESY correlations in compounds **3** and **4**.

Compound **3** was obtained as a light-yellow gum. The molecular formula of compound **3** was determined to be C_21_H_33_NO_5_ based on the high-resolution electrospray ionization mass spectrum (HRESIMS) (*m*/*z* [M+H]^+^ 380.2435, calcd. for C_21_H_34_NO_5_^+^, 380.2431), accounting for six degrees of unsaturation (Figure S15). The ^1^H NMR spectrum (Figure S16, Table 2) of compound **3** showed signals for four olefinic protons at *δ*_H_ 5.91 (1H, d, *J* = 15.0 Hz), 7.00 (1H, dd, *J* = 15.0, 10.5 Hz), 6.17 (1H, dd, *J* = 15.0, 10.5 Hz), and 6.08 (1H, dt, *J* = 15.0, 7.0 Hz); ten methylene signals at *δ*_H_ 2.12 (dt, *J* = 7.0, 7.0 Hz), 1.37 (m), 1.27 (m), 1.25 (m), 1.24 (m), 1.26 (m), 1.12 (m), 2.18 (dd, *J* = 15.0, 6.0 Hz), 1.99 (dd, *J* = 15.0, 8.0 Hz), 3.90 (dd, *J* = 9.5, 4.5 Hz), 3.59 (dd, *J* = 9.5, 1.5 Hz), 2.04 (m) and 1.83 (m), and 3.75 (m); four sp^3^ methine protons at *δ*_H_ 1.79 (m), 4.10, (m), 2.66 (ddd, *J* = 9.5, 5.0, 4.5 Hz), and 5.67 (d, *J* = 5.0 Hz’); and one methyl signal at 0.87 (d, *J* = 7.0 Hz). The ^13^C NMR and HSQC spectra (Figures S17 and S18) of compound **3** displayed 21 carbon signals, including four sp^2^ methines at *δ*_C_ 122.8, 139.6, 128.5, and 142.0; four sp^3^ methines at 29.6, 56.0, 49.1, and 108.2; ten sp^3^ methylenes at *δ*_C_ 32.2, 28.3, 28.6, 29.0, 26.3, 36.0, 41.4, 72.3, 29.9, and 67.2; one methyl at *δ*_C_ 19.6; and two carboxyl groups at *δ*_C_ 165.0 and 174.0. The ^1^H-^1^H COSY correlations (Figure S19 and Figure 4) revealed the substructures of C-3/C-4/C-5/C-6/C-7/C-8/C-9, C-11/C-12/C-13/C-14(C-16), and C-2′/C-3′(N-1)/C-4′(C-8′)/C-5′/C-6′. In the HMBC spectrum (Figure S20 and Figure 4), the long-range correlations from H-3 (*δ*_H_ 4.10) to C-2 (*δ*_C_ 165.0) and C-5 (*δ*_C_ 128.5), and from H-4 (*δ*_H_ 7.00) to C-2 (*δ*_C_ 165.0) and C-3 (*δ*_C_ 122.8), indicated the carboxyl group of C-2. The HMBC correlation from H-14 (*δ*_H_ 2.18 and 1.99) to C-15 (*δ*_C_ 174.0) revealed the carboxyl group of C-15. The bis-tetrahydrofuran moiety was confirmed by HMBC correlations from H-2′ (*δ*_H_ 3.90 and 3.59) and H-6′ (3.75) to C-8′ (*δ*_C_ 108.2), and from H-8′ (*δ*_H_ 5.67) to C-2′ (*δ*_C_ 72.3) and C-6′ (*δ*_C_ 67.2). The HMBC correlations from H-NH (*δ*_H_ 8.30) to C-2, C-3, and C-3′ (*δ*_C_ 56.0) revealed the presence of amide. The geometric configurations of the double bonds C-3/C-4/C-5/C-6 were determined by an analysis of the coupling constants [*δ*_H_ 5.91 (1H, d, *J* = 15.0 Hz, H-3), 7.00 (1H, dd, *J* = 15.0, 10.5 Hz, H-4), 6.17 (1H, dd, *J* = 15.0, 10.5 Hz, H-5), and 6.08 (1H, dt, *J* = 15.0, 7.0 Hz, H-6)]. Therefore, the planar structure of compound **3** was determined. In the ROESY spectrum, the correlation between H-3′ (*δ*_H_ 4.10) and H-5b’ (*δ*_H_ 1.83) revealed the same orientations of H-3′ and H-5b’. The coupling constant of H-3′ and H-4′ (*J* = 5.0 Hz), together with the ROESY correlations (Figure S21 and Figure 4) from H-4′ to H-5′a and H-8′, indicated the relative configurations of C-3′, C-4′, and C-8′. Compound **3** gave a negative optical rotation which shared the same orientation as that of (3*S*,3a*S*,6a*S*)-5-oxohexahydrofuro [2,3-b]furan-3-carboxylic acid [29], revealing the same configuration for the bis-tetrahydrofuran moiety. The configuration of C-13 was not defined. Therefore, the structure of compound **3** was determined and it was named penirubenamide A.

Compound **4** was obtained as a light-yellow gum. The molecular formula of compound **4** was determined to be C_21_H_35_NO_4_ based on the high-resolution electrospray ionization mass spectrum (HRESIMS) (*m*/*z* [M + H]^+^ 366.2640, calcd. for C_21_H_35_NO_5_^+^, 366.2639), accounting for five degrees of unsaturation (Figure S22). The ^1^H and ^13^C NMR spectra (Figures S23 and S24, Table 3) of compound **4** showed similar signals to those of compound **3**. A detailed analysis of the 2D NMR data (Figures S25–S27 and Figure 4) revealed that the carboxyl of C-15 (*δ*_C_ 174.0) in compound **3** was replaced by hydroxylmethylene (*δ*_C_ 58.8, *δ*_H_ 3.40). These data were also confirmed by ^1^H-^1^H COSY correlations between H_2_-14 (*δ*_H_ 1.43 and 1.19) and H_2_-15 (*δ*_H_ 3.40), and HMBC correlations from H_2_-15 to C-13 (*δ*_C_ 28.9) and C-14 (*δ*_C_ 39.9). The geometric configurations of double bonds C-3/C-4/C-5/C-6 were confirmed by a detailed analysis of the coupling constants [*δ*_H_ 5.91 (d, *J* = 15.0 Hz, H-3), 7.00 (dd, *J* = 15.0, 10.5 Hz, H-4), 6.18 (dd, *J* = 15.0, 10.5 Hz, H-5), and 6.08 (dt, *J* = 15.0, 7.0 Hz, H-6)]. The coupling constant of H-8′ (*δ*_H_ 5.66) for compound **4** was the same as that of compound **3** (*J* = 5.0 Hz), which indicated the same orientation of H-4′ (*δ*_H_ 2.66) and H-8′. The ROESY correlation between H-3′ (*δ*_H_ 4.10) and H-5b’ (*δ*_H_ 1.82), from H-4′ to H-5′a (*δ*_H_ 2.04), revealed the relative configurations of C-3′, C-4′, and C-8′. The configuration of C-13 was not defined. Therefore, the structure of **4** was determined and it was named penirubenamide B.

The known compounds were identified as penimeroterpenoid A (**5**) [30], 2,4′-linked secalonic acid (**6**) [31], dihydrocitreohybridonol (**7**) [32], 3-deacetylated andrastin A (**8**) [33], citreohybridonol (**9**) [34], penicyclone A **10**) [35], penicyclone D (**11**) [35], penicyclone E (**12**) [35], and peniginsengin A (**13**) [36].

### 3.3. Antibacterial Activities of the Isolated Compounds

All the isolated compounds were evaluated for their antimicrobial activities against *C. albicans*, *S. aureus*, and *E. coli*, as well as for synergistic antifungal activity against *C. albicans*. None of the compounds showed inhibitory effects on *E. coli*. Compound **6** displayed antibacterial activity against *S. aureus*, with an MIC value of 3.125 μg/mL. All the tested compounds did not show antifungal activity at a concentration of 200 μg/mL (MIC for rapamycin is 0.5 μg/mL). To explore the potential of these compounds, the synergistic antifungal activity in combination with rapamycin was determined. Compounds **1** and **2** exhibited synergistic antifungal activity against *C. albicans* at 12.5 and 50 μg/mL, respectively, when combined with 0.0625 μg/mL rapamycin.

## 4. Conclusions

**Figure 5:**
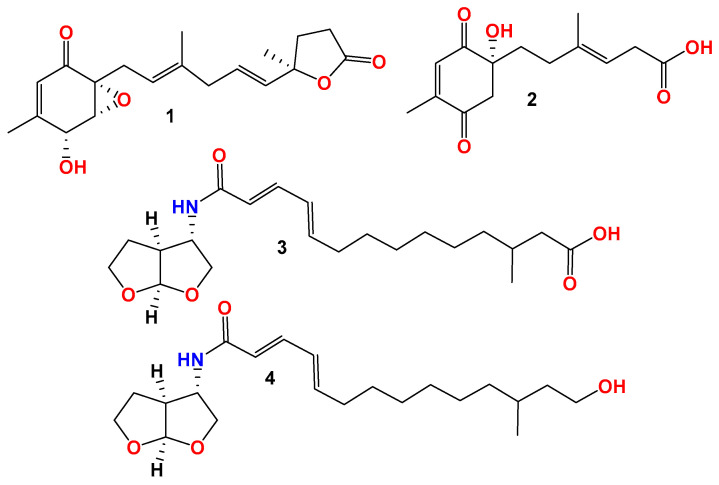
Structures of compounds **1**–**4**.

During the chemical investigation of new chemical entries from marine-derived fungi, two new polyketide derivatives, penirubenones A and B (compounds **1** and **2**), together with two naturally rare amino-bis-tetrahydrofuran derivatives, penirubenamides A and B (compounds **3** and **4**) (Figure 5), were isolated from the marine-derived *Penicillium rubens* BTBU20213035. The planar structures and relative configurations were characterized by a detailed HRESIMS and ^1^D and ^2^D NMR analyses. Furthermore, the absolute configurations were determined by calculations of electronic circular dichroism (ECD) and NMR, as well as by comparison with the reported data. Compounds **1** and **2** share the carbon skeleton of ambuic acid analogues, which have been identified from marine-derived fungus of *Penicillium* [35]. The carbon skeleton of compounds **3** and **4** was reported as a natural product for the first time, to the best of our knowledge. Other classes of compounds were also identified from this marine-derived fungus, such as secalonic acid analogue (**6**), andrastin-type meroterpenoids (**5**, **7**–**9**), and ambuic acid analogues (**10**–**13**). These results revealed the high chemical diversity of *Penicillium rubens* BTBU20213035. Further investigation using the one strain–many compounds (OSMAC) method will lead to the identification of more new secondary metabolites. In the antimicrobial evaluation, compound **6** inhibited the growth of *S aureus*, with an MIC value of 3.125 μg/mL. Compound **6** has also been reported to have cytotoxic activity against HepG2 cells [31]. Our study broadened the application of the secalonic acid analogue. Compounds **1** and **2** showed synergistic antifungal activity when combined with rapamycin. Synergisms can typically delay the development of drug resistance, so, detailed investigations on the drug resistance development of compounds **1** and **2** will be performed in future studies.

## Figures and Tables

**Table 1:** ^1^H (500 MHz) and ^13^C (125 MHz) NMR data of **1** and **2**.

Position	1 (CD_3_OD)		2 (CDCl_3_)	
	*δ*_C_	*δ*_H_ (mult, *J* in Hz)	*δ*_C_	*δ*_H_ (mult, *J* in Hz)
1	195.8, C		201.4, C	
2	123.6, CH	5.72 (t, 1.5)	134.4, CH	6.70 (m)
3	159.6, C		152.5, C	
4	68.1, CH	4.51 (m)	196.1, C	
5a	60.7, CH	3.64 (d, 3.0)	51.3, CH_2_	3.17 (d, 16.0)
5a				2.92 (d, 16.0)
6	61.7, C		77.9, C	
7	27.4, CH_2_	2.44 (dd, 7.0, 15.0) 2.74 (overlap)	38.6, CH_2_	1.87 (m) 1.70 (m)
8a	119.6, CH	5.11 (m)	33.0, CH_2_	2.12 (m)
8b				1.87 (m)
9	138.6, C		138.4, C	
10	43.0, CH_2_	2.74 (overlap)	116.2, CH	5.26 (t, 7.0)
11	128.9, CH	5.62 (overlap)	33.3, CH_2_	3.05 (d, 7.0)
12	135.1, CH	5.65 (overlap)	177.4, C	
13	87.2, C		16.4, CH_3_	2.04 (d, 1.5)
14	34.9, CH_2_	2.22 (m) 2.12 (m)	16.6, CH_3_	1.58 (s)
15	29.8, CH_2_	2.57 (m)		
16	179.6, C			
17	20.3, CH_3_	1.98 (t, 1.0)		
18	16.5, CH_3_	1.64 (br s)		
19	26.6, CH_3_	1.48 (d, 1.5)		

**Table 2:** ^1^H (500 MHz) and ^13^C (125 MHz) NMR data of **3** and **4** (DMSO-*d*_6_).

Pos.	3		4	
	*δ*_C_	*δ*_H_ (*J* in Hz)	*δ*_C_	*δ*_H_ (*J* in Hz)
1-NH		8.30 (d, 7.5)		8.30 (d, 6.5)
2	165.0, C		165.0, C	
3	122.8, CH	5.91 (d, 15.0)	122.8, CH	5.91 (d, 15.0)
4	139.6, CH	7.00 (dd, 15.0, 10.5)	139.6, CH	7.00 (dd, 15.0, 10.5)
5	128.5, CH	6.17 (dd, 15.0, 10.5)	128.5, CH	6.18 (dd, 15.0, 10.5)
6	142.0, CH	6.08 (dt, 15.0. 7.0)	142.1, CH	6.08 (dt, 15.0, 7.0)
7	32.2, CH_2_	2.12 (dt, 7.0, 7.0)	32.3, CH_2_	2.12 (dt, 7.0, 6.5)
8	28.3, CH_2_	1.37 (m)	28.4, CH_2_	1.37 (m)
9	28.6, CH_2_	1.27 (m)	28.6, CH_2_	1.27 (m)
10	29.0, CH_2_	1.25 (m)	29.2, CH_2_	1.26 (m)
11	26.3, CH_2_	1.24 (m)	26.3, CH_2_	1.24 (m)
12a	36.0, CH_2_	1.26 (m)	36.6, CH_2_	1.24 (m)
12b		1.12 (m)		1.06 (m)
13	29.6, CH	1.79 (m)	28.9, CH_2_	1.49 (m)
14	41.4, CH_2_	2.18 (dd, 15.0, 6.0)	39.9, CH_2_	1.43 (m)
		1.99 (dd, 15.0, 8.0)		1.19 (m)
15	174.0, C		58.8, CH_2_	3.40 (m)
16	19.6, CH_3_	0.87 (d, 7.0)	19.6, CH_3_	0.82 (d, 6.5)
2′	72.3, CH_2_	3.90 (dd, 9.5, 4.5)	72.3, CH_2_	3.91 (dd, 9.5, 5.0)
		3.59 (dd, 9.5, 1.5)		3.59 (dd, 9.5, 1.5)
3′	56.0, CH	4.10 (m)	56.0, CH	4.10 (m)
4′	49.1, CH	2.66 (ddd, 9.5, 5.0, 4.5)	49.1, CH	2.66 (m)
5′a	29.9, CH_2_	2.04 (m, 5′a)	29.8, CH_2_	2.04 (m, 5′a)
5′b		1.83 (m, 5′b)		1.82 (m, 5′b)
6′	67.2, CH_2_	3.75 (m)	67.2, CH_2_	3.74 (m)
8′	108.2, CH	5.67 (d, 5.0)	108.2, CH	5.66 (d, 5.0)

**Table 3:** Comparison of experimental and calculated ^13^C NMR for compound **1**.

Position	1	1-1	1-2
1	195.8	191.8	191.8
2	123.6	121.6	120.0
3	159.6	159.2	159.1
4	68.1	68.6	68.6
5	60.7	59.1	59.5
6	61.7	58.6	58.5
7	27.4	30.5	30.6
8	119.6	119.3	119.1
9	138.6	133.2	133.4
10	43.0	43.1	46.2
11	128.9	125.5	124.1
12	135.1	130.9	130.7
13	87.2	83.5	83.3
14	34.9	35.4	35.4
15	29.8	29.1	29.1
16	179.6	172.5	172.5
17	20.3	21.9	21.9
18	16.5	17.3	17.1
19	26.6	27.7	27.4
R2		0.9999	0.9988
MAE		2.29	2.60
MaxErr		7.09	7.12

## Data Availability

The original contributions presented in the study are included in the article/Supplementary Material, further inquiries can be directed to the corresponding authors.

## Supplementary Materials

The following supporting information can be downloaded at: https://www.mdpi.com/article/10.3390/jof10060424/s1, Figures S1–S7: HRESIMS, ^1^H, ^13^C, HSQC, ^1^H–^1^H COSY, HMBC, and ROESY spectra for compound **1**; Figures S8–S14: HRESIMS, ^1^H, ^13^C, HSQC, ^1^H–^1^H COSY, HMBC, and ROESY spectra for compound **2**; Figures S15–S21: HRESIMS, ^1^H, ^13^C, HSQC, ^1^H–^1^H COSY, HMBC, and ROESY spectra for compound **3**; Figures S22–S28: HRESIMS, ^1^H, ^13^C, HSQC, ^1^H–^1^H COSY, HMBC, and ROESY spectra for compound **4**; Figure S29: Structures of compounds **5**–**13**.
