# Supplementary material for: Secondary Metabolites from Marine-Derived Fungus Penicillium rubens BTBU20213035
Source: J Fungi (Basel). 2024 Jun 16;10(6):424. doi: 10.3390/jof10060424 (PMC11204949; doi:10.3390/jof10060424)
Supplement: Supplementary file 1 [file jof-10-00424-s001.zip › jof-3044448-supplementary.pdf]

SUPPLEMENTARY MATERIAL

Secondary Metabolites from Marine-Derived Fungus *Penicillium rubens* BTBU20213035

Xiuli Xu [1], Yifei Dong [2], Jinpeng Yang [1], Long Wang [3], Linlin Ma [4], Fuhang Song [2,*], Xiaoli Ma [5,*]

[1] Key Laboratory of Marine Mineral Resources and Polar Geology, Ministry of Education, School of Ocean Sciences, China University of Geosciences, Beijing 100083, China
[2] Key Laboratory of Geriatric Nutrition and Health, Ministry of Education of China, School of Light Industry Science and Engineering, Beijing Technology and Business University, Beijing 100048, China
[3] State Key Laboratory of Mycology, Institute of Microbiology, Chinese Academy of Sciences, Beijing 100101, China
[4] Griffith Institute for Drug Discovery, School of Environment and Science, Griffith University, Brisbane 4111, Australia
[5] School of Chemistry and Chemical Engineering, North Minzu University, Yinchuan 750021, China
* Correspondence: songfuhang@btbu.edu.cn (F.S.); mxl@nmu.edu.cn (X.M.)

Table of Contents

Figure S1. HRESIMS spectrum for **1** 3

Figure S2. ^{1}H NMR spectrum (500 MHz, CD_3OD) of **1** 3

Figure S3. ^{13}C NMR spectrum (125 MHz, CD_3OD) of **1** 4

Figure S4. HSQC spectrum (500 MHz, CD_3OD) of **1** 4

Figure S5. ^{1}H -^{1}H COSY spectrum (500 MHz, CD_3OD) of **1** 5

Figure S6. HMBC spectrum (500 MHz, CD_3OD) of **1** 5

Figure S7. ROESY spectrum (500 MHz, CD_3OD) of **1** 6

Figure S8. HRESIMS spectrum for **2** 6

Figure S9. ^{1}H NMR spectrum (500 MHz, $CDCl_3$) of **2** 7

Figure S10. ^{13}C NMR spectrum (125 MHz, $CDCl_3$) of **2** 7

Figure S11. HSQC spectrum (500 MHz, $CDCl_3$) of **2** 8

Figure S12. ^{1}H -^{1}H COSY spectrum (500MHz, $CDCl_3$) of **2** 8

Figure S13. HMBC spectrum (500MHz, $CDCl_3$) of **2** 9

Figure S14. ROESY spectrum (500MHz, $CDCl_3$) of **2** 9

Figure S15. HRESIMS spectrum for **3** 10

Figure S16. ^{1}H NMR spectrum (500 MHz, DMSO-d_6) of **3** 10

Figure S17. ^{13}C NMR spectrum (125 MHz, DMSO-d_6) of **3** 11

Figure S18. HSQC spectrum (500 MHz, DMSO-d_6) of **3** 11

Figure S19. ^{1}H -^{1}H COSY spectrum (500MHz, DMSO-d_6) of **3** 12

Figure S20. HMBC spectrum (500 MHz, DMSO-d_6) of **3** 12

Figure S21. ROESY spectrum (500 MHz, DMSO-d_6) of **3** 13

Figure S22. HRESIMS spectrum for **4** 13

Figure S23. ^{1}H NMR spectrum (500 MHz, DMSO-d_6) of **4** 14

Figure S24. ^{13}C NMR spectrum (125 MHz, DMSO-d_6) of **4** 14

Figure S25. HSQC spectrum (500 MHz, DMSO-d_6) of **4** 15

Figure S26. ^{1}H -^{1}H COSY spectrum (500MHz, DMSO-d_6) of **3** 15

Figure S27 HMBC spectrum (500 MHz, DMSO-d_6) of **4** 16

Figure S28. ROESY spectrum (500 MHz, DMSO-d_6) of **4** 16
Figure S29. The structures of compounds **5-13** 17

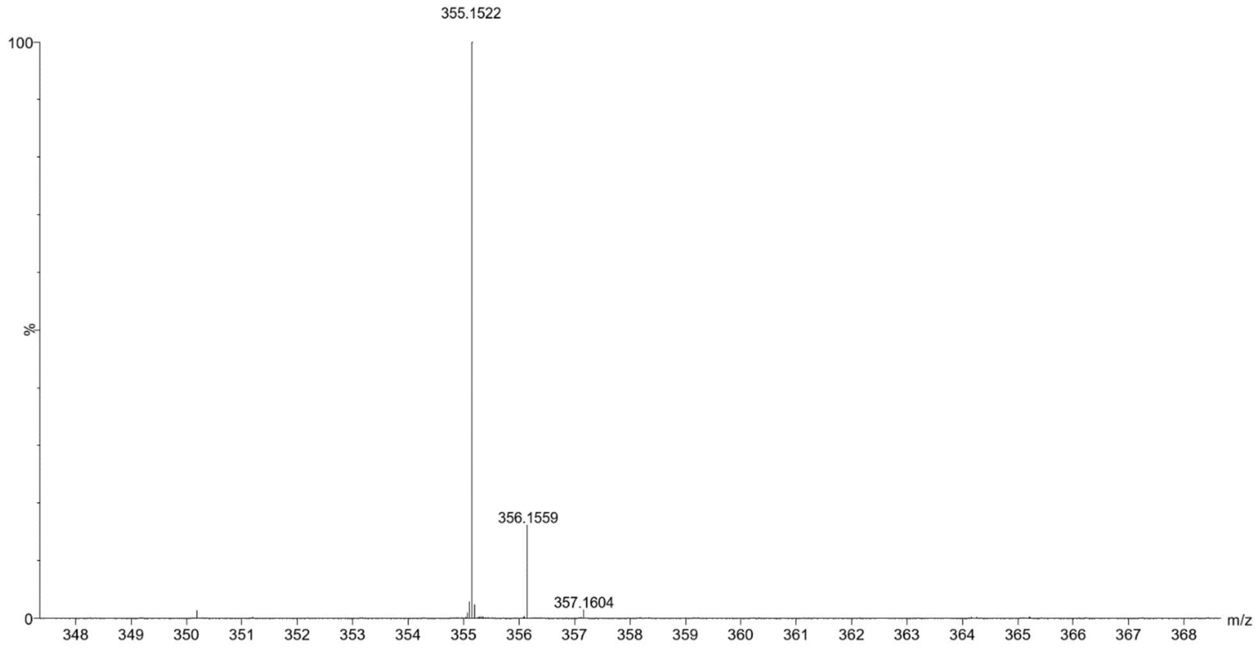

Figure S1. HRESIMS spectrum for **1**

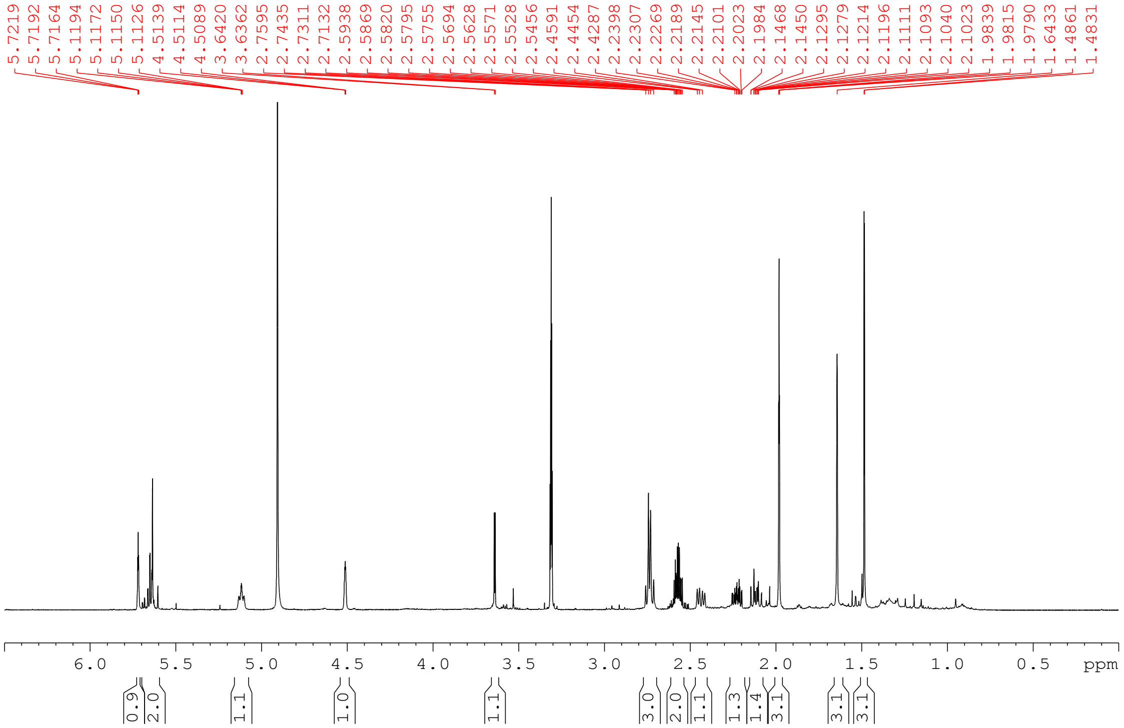

Figure S2. ^{1}H NMR spectrum (500 MHz, CD_3OD) of **1**

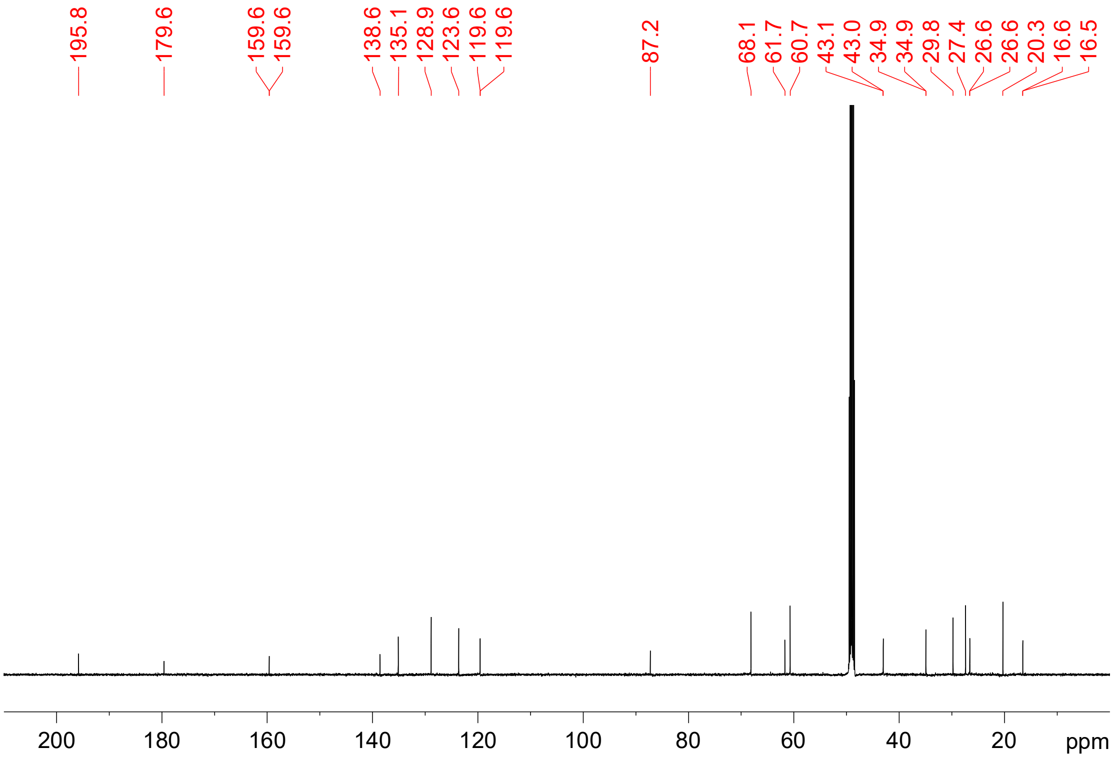

Figure S3. ^{13}C NMR spectrum (125 MHz, CD_3OD) of **1**

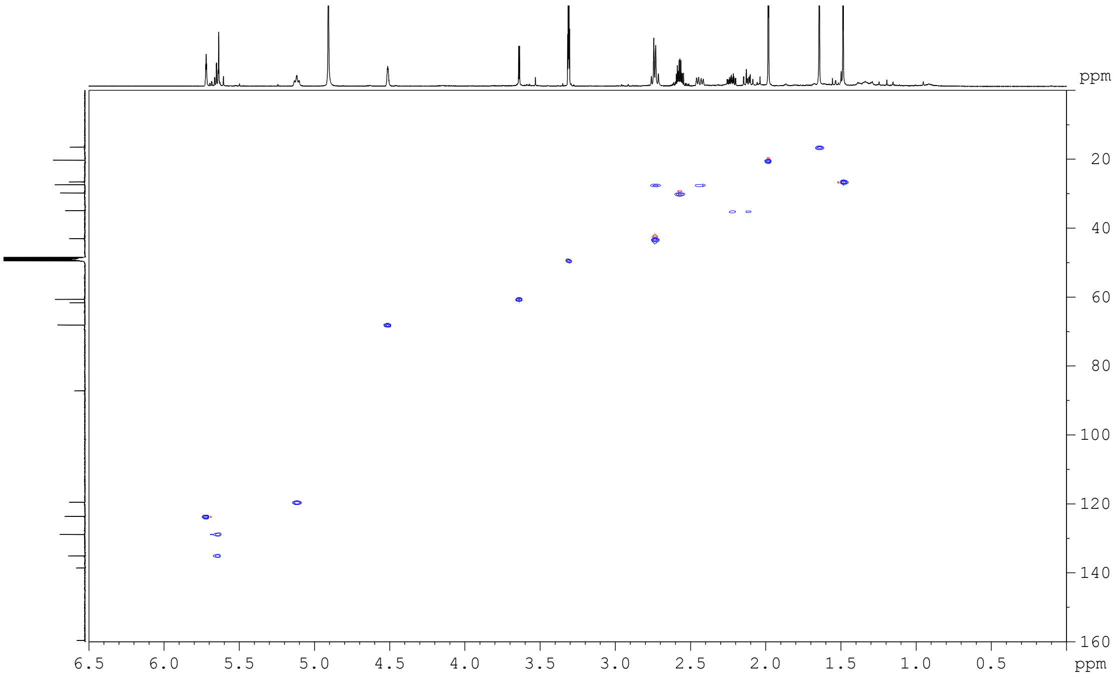

Figure S4. HSQC spectrum (500 MHz, CD_3OD) of **1**

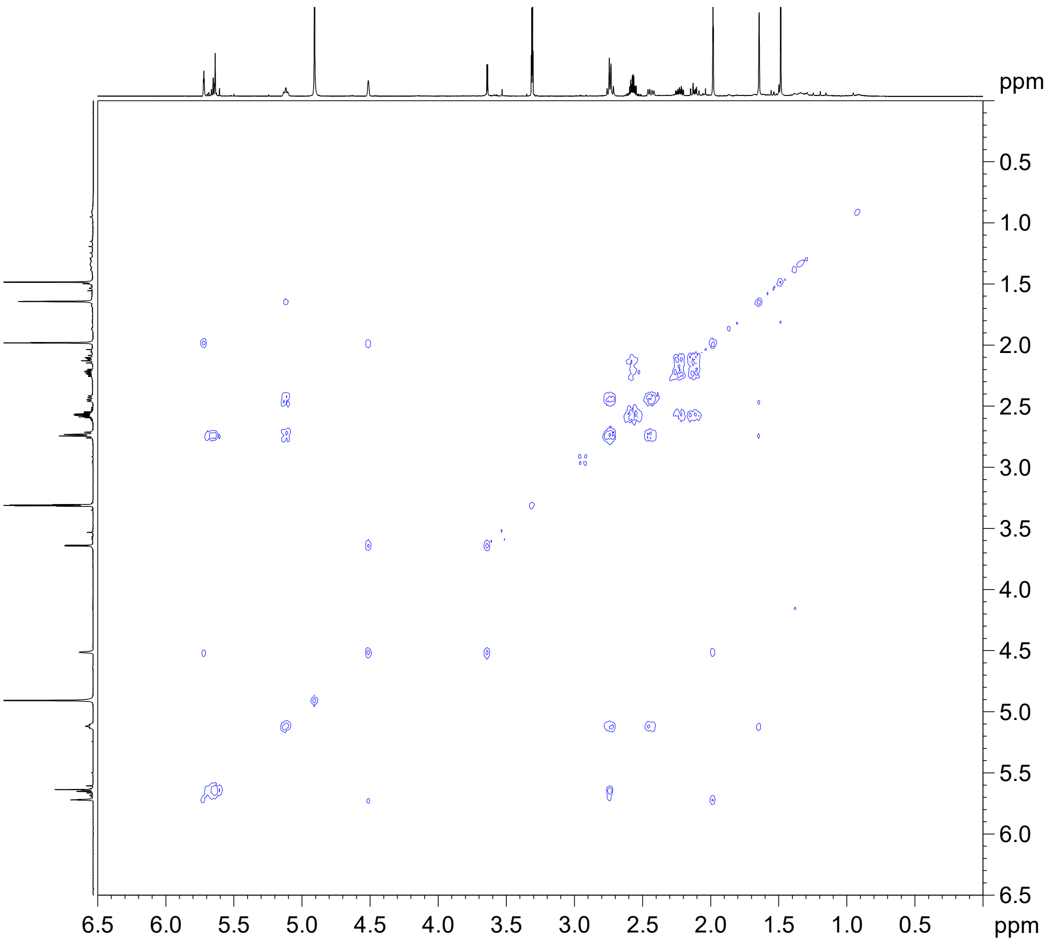

Figure S5. ^{1}H -^{1}H COSY spectrum (500 MHz, CD_3OD) of **1**

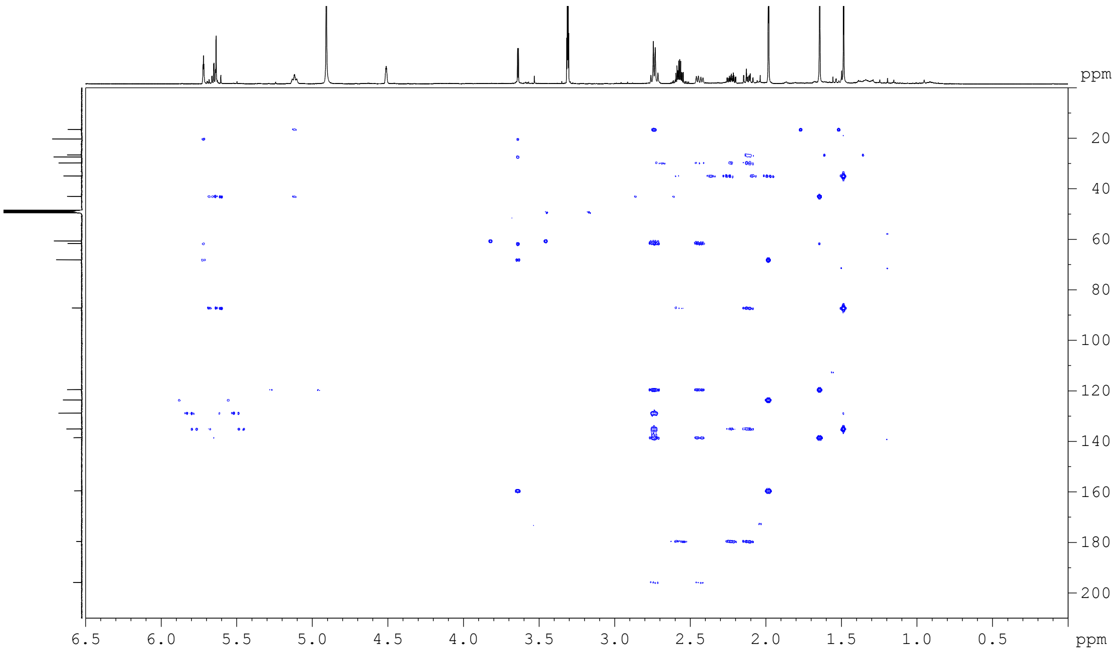

Figure S6. HMBC spectrum (500 MHz, CD_3OD) of **1**

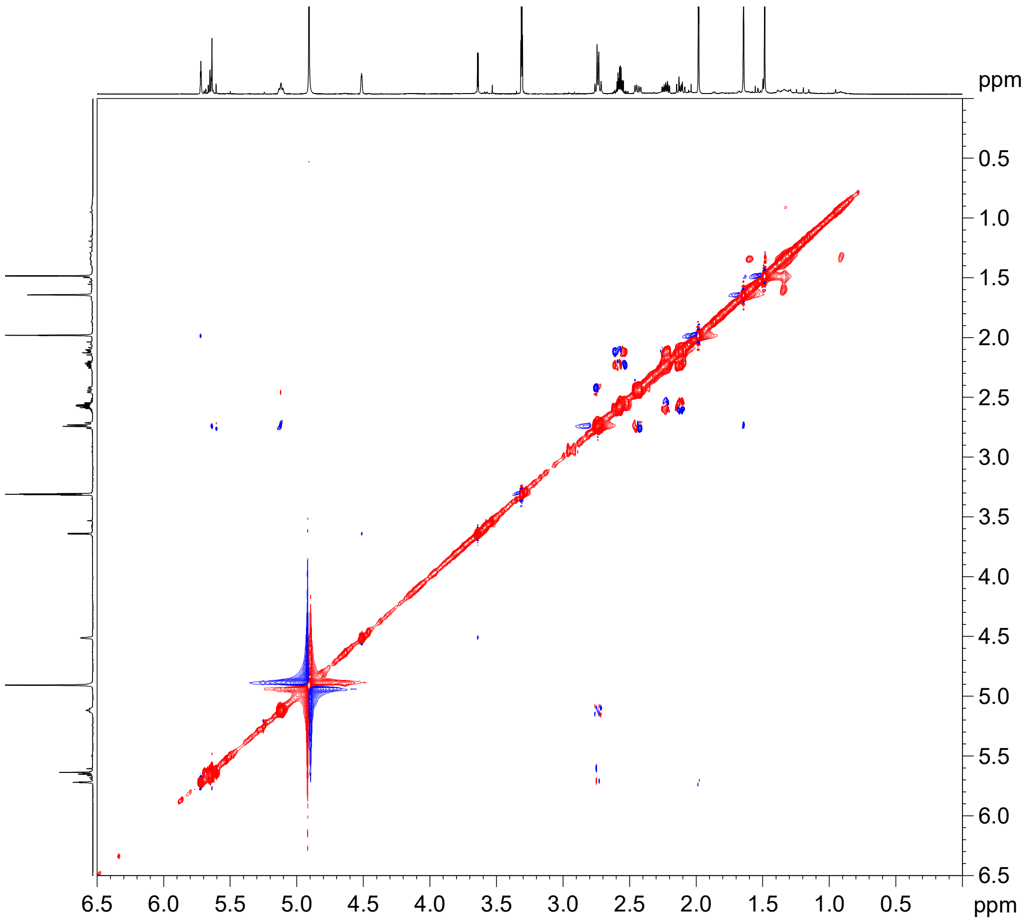

Figure S7. ROESY spectrum (500 MHz, CD_3OD) of **1**

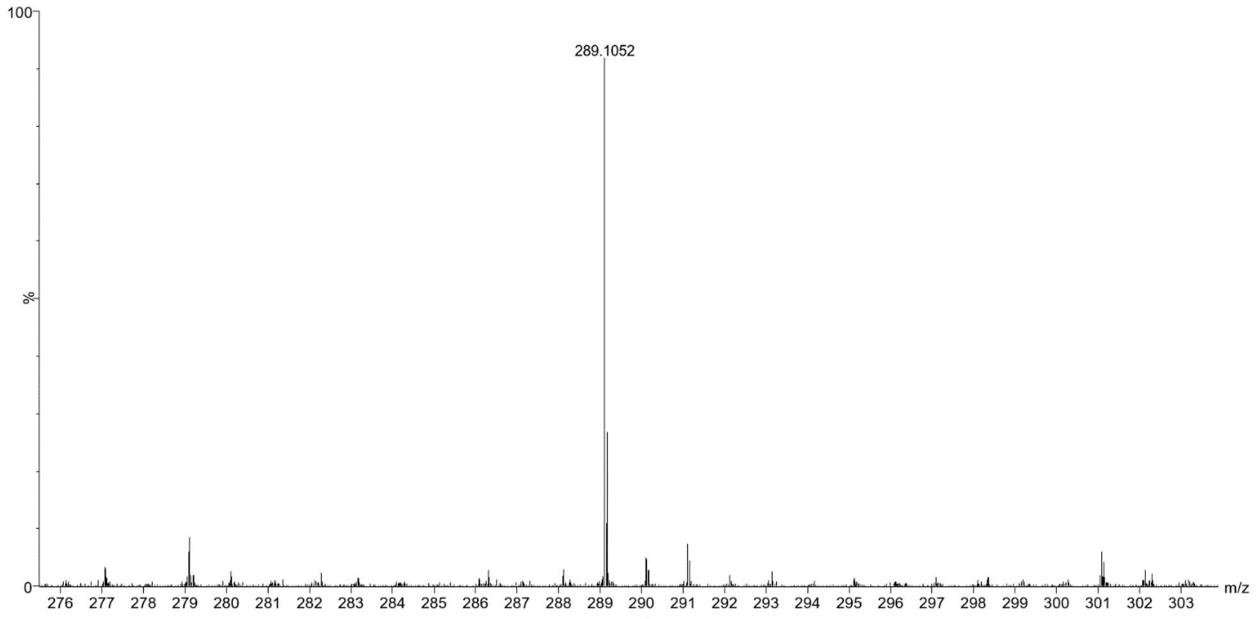

Figure S8. HRESIMS spectrum of **2**

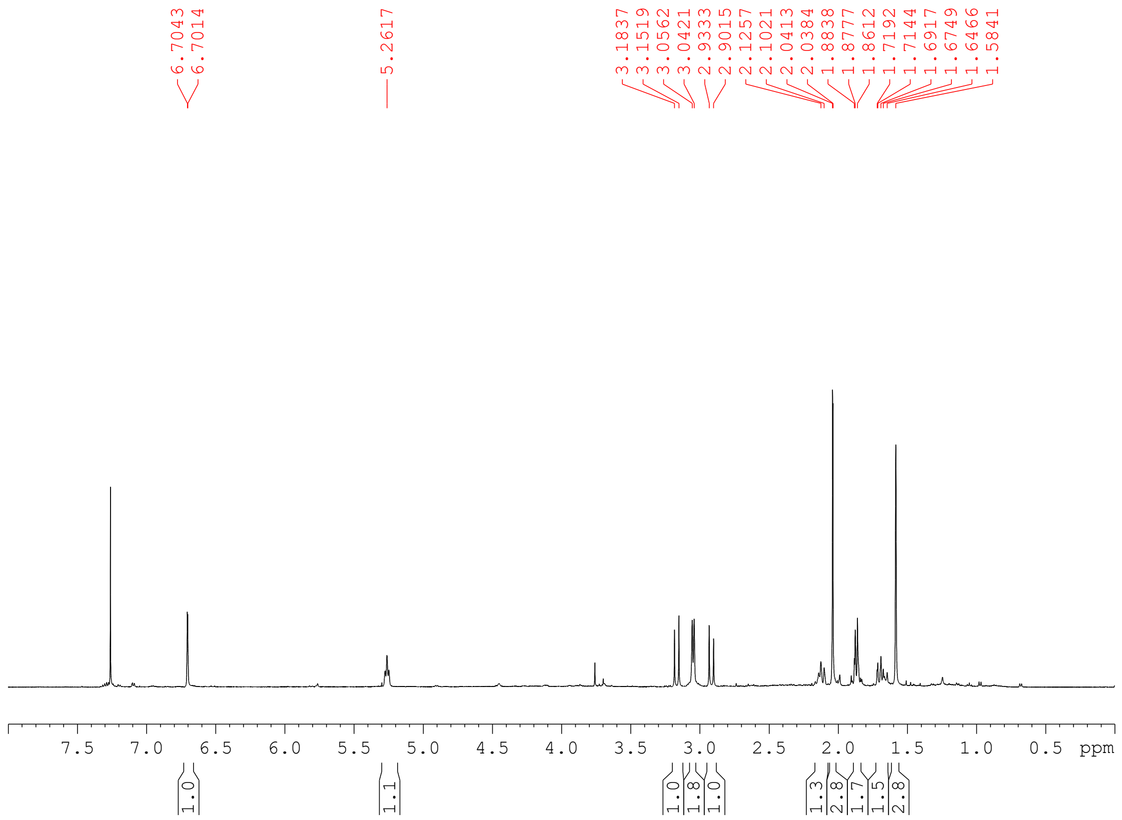

Figure S9. ¹H NMR spectrum (500 MHz, $CDCl_3$) of **2**

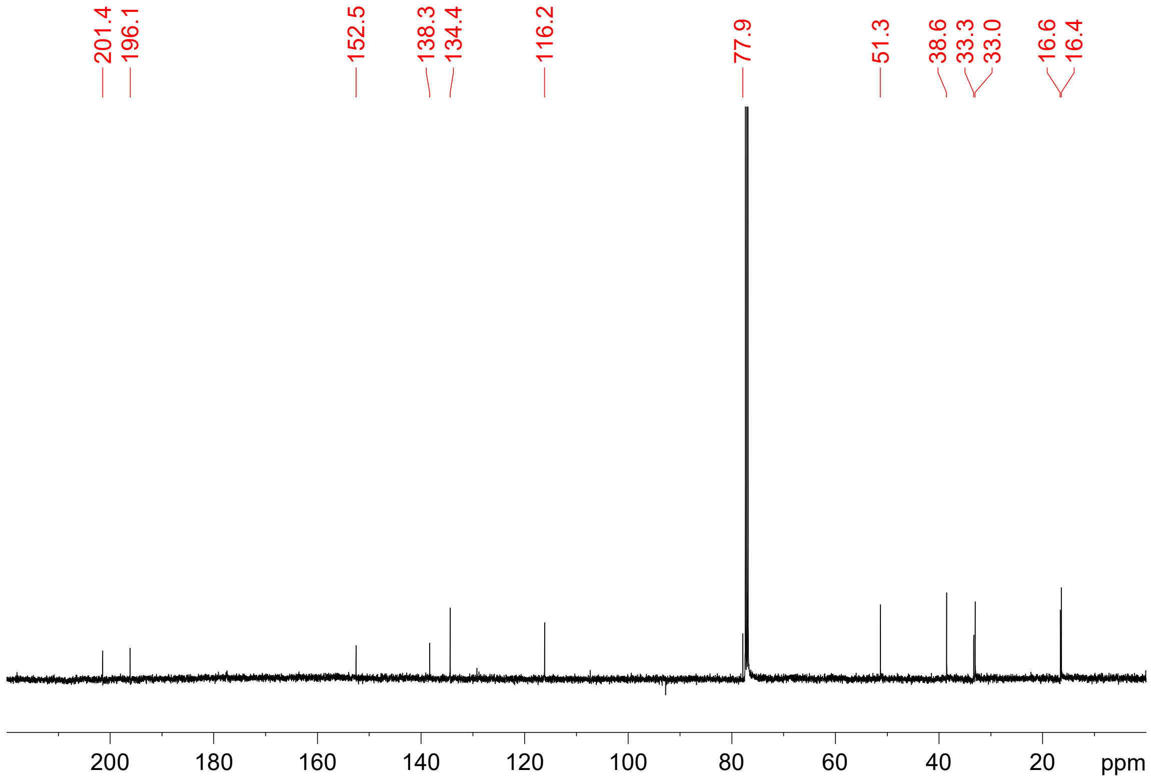

Figure S10. ¹³C NMR spectrum (125 MHz, $CDCl_3$) of **2**

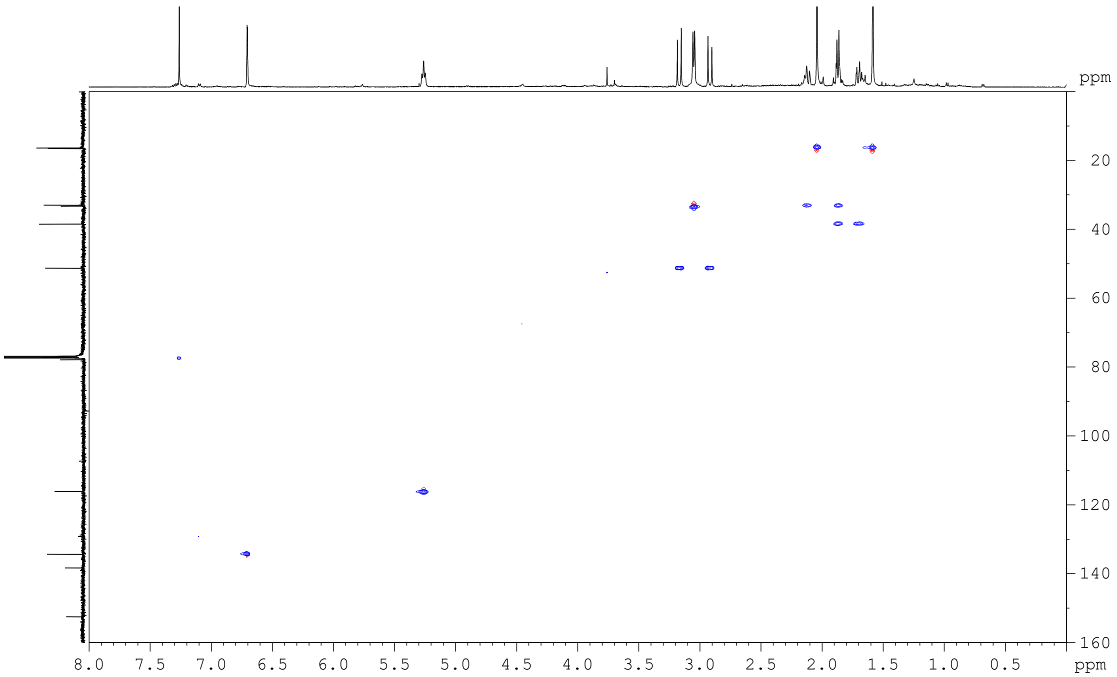

Figure S11. HSQC spectrum (500 MHz, $CDCl_3$) of **2**

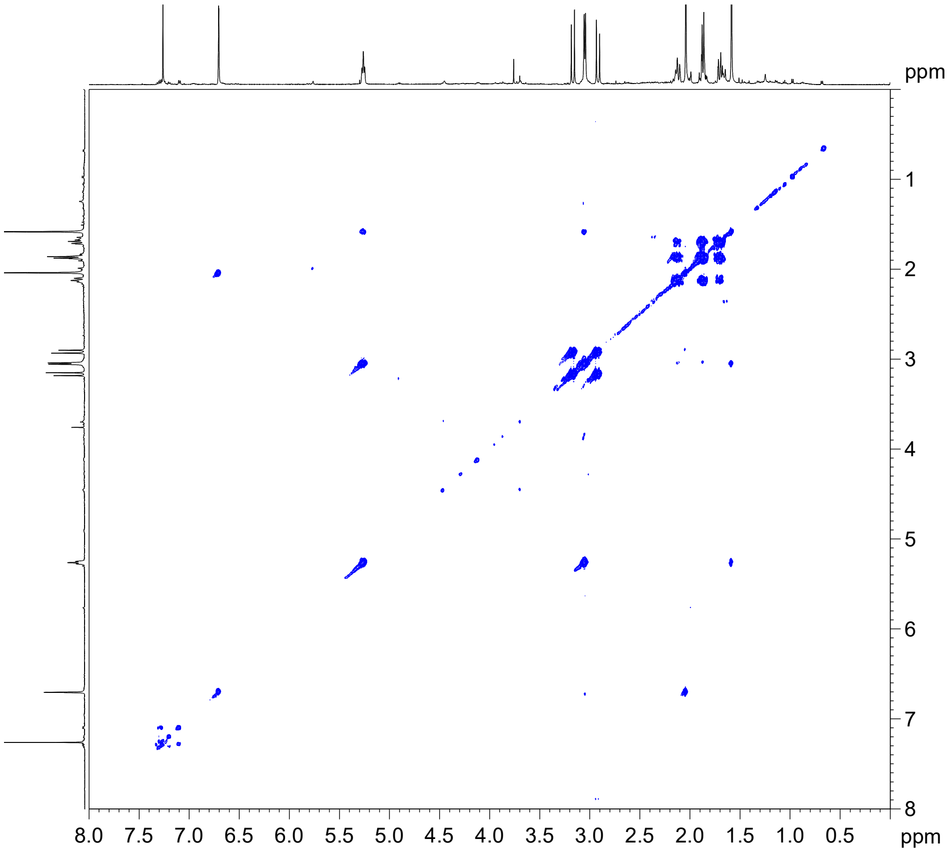

Figure S12. ^1H -^1H COSY spectrum (500MHz, $CDCl_3$) of **2**

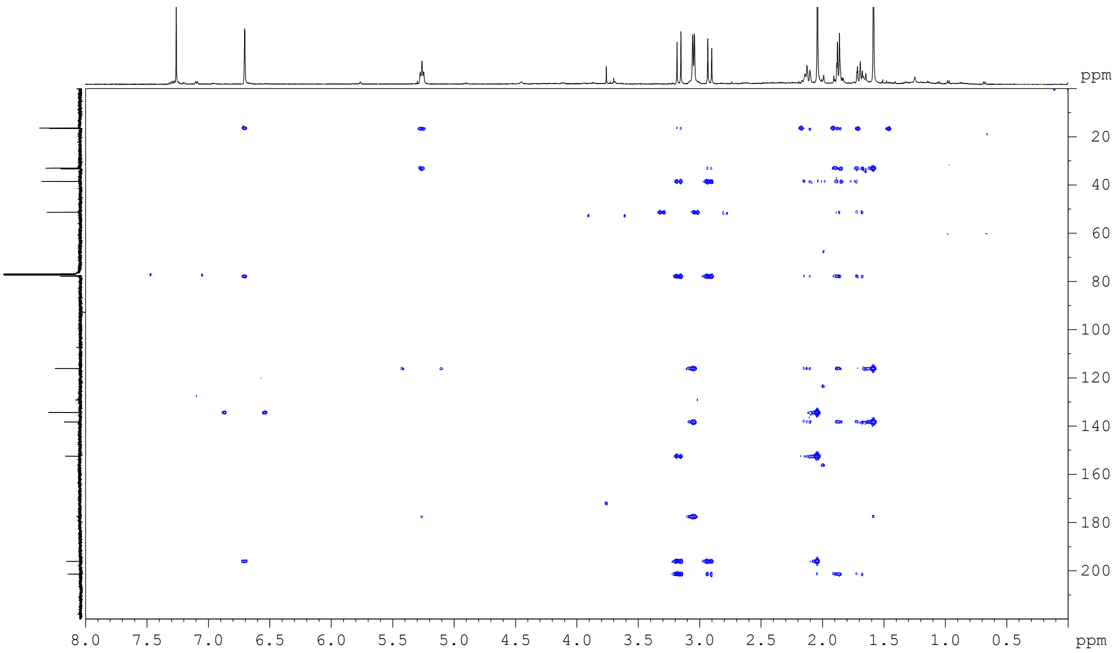

Figure S13. HMBC spectrum (500 MHz, $CDCl_3$) of **2**

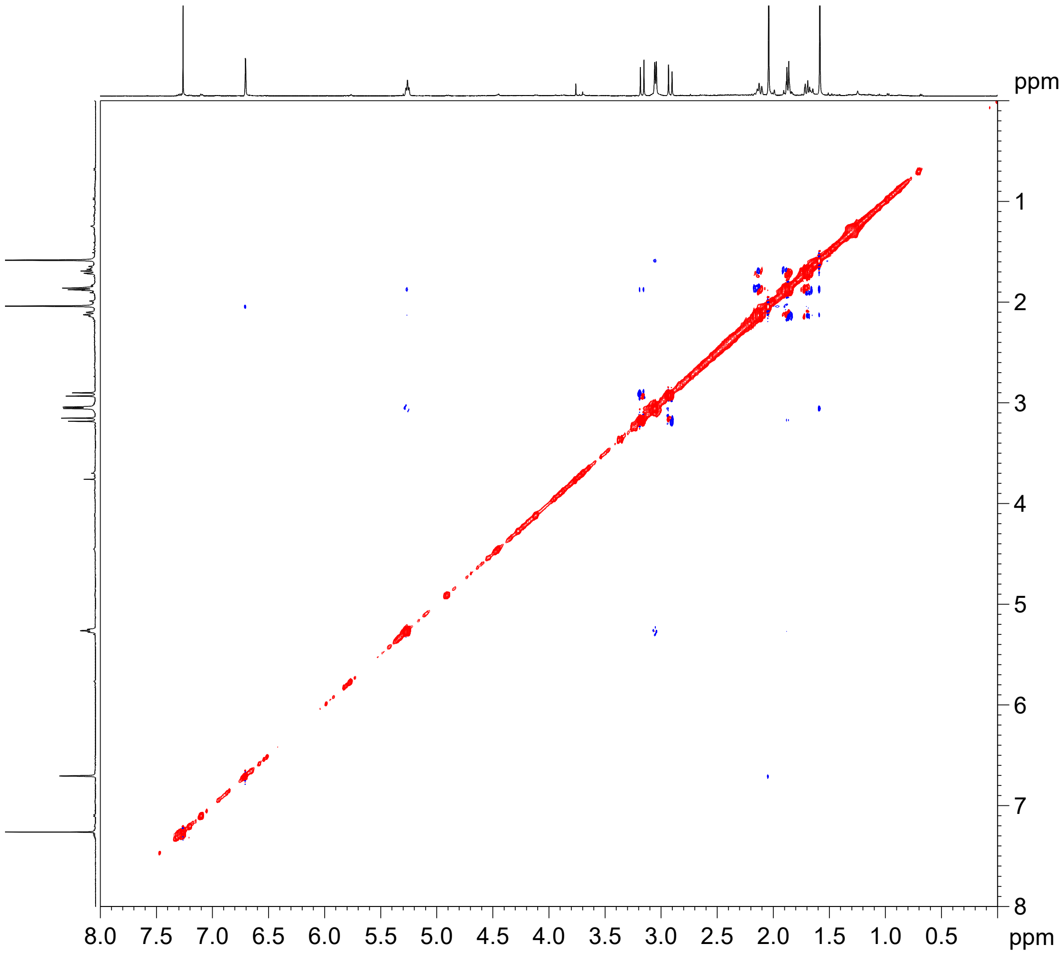

Figure S14. ROESY spectrum (500 MHz, $CDCl_3$) of **2**

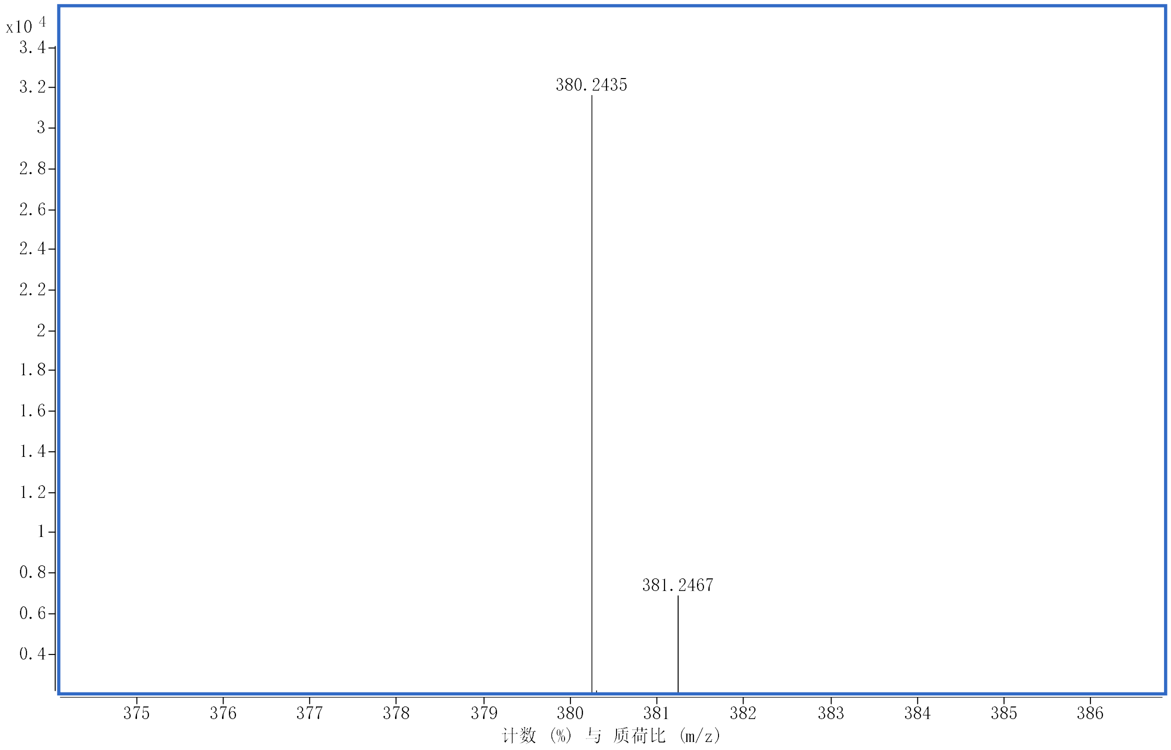

Figure S15. HRESIMS spectrum of **3**

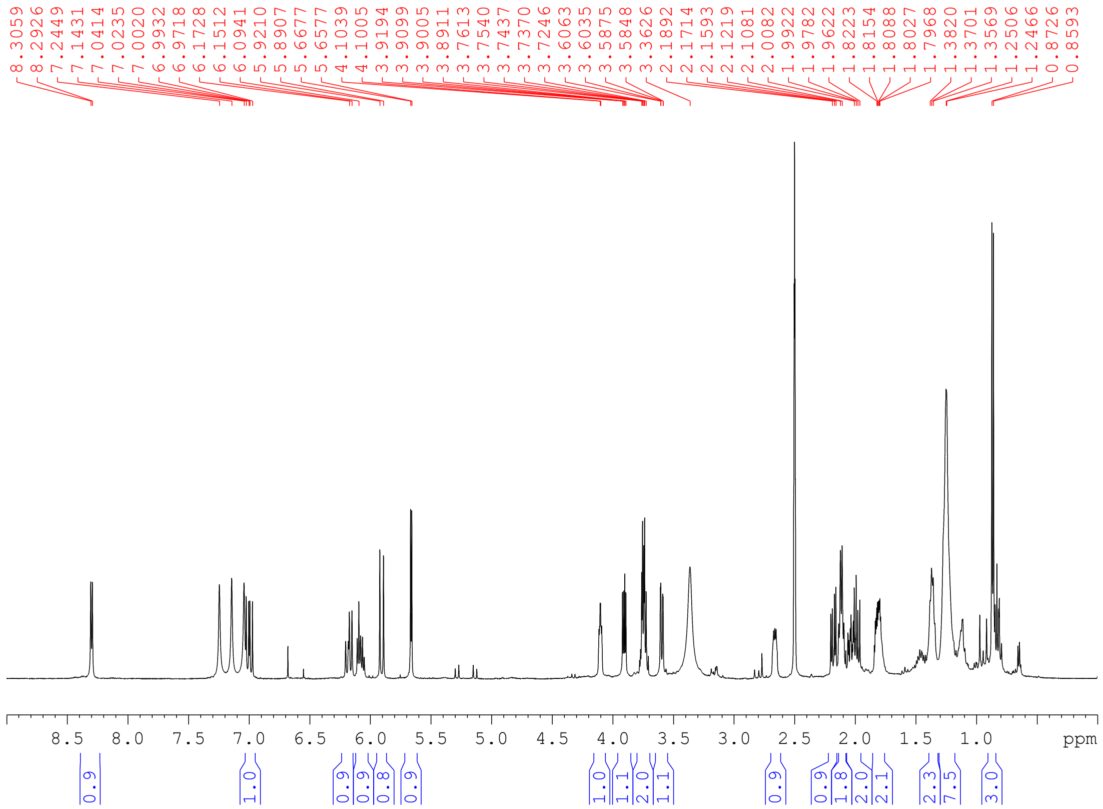

Figure S16. ^{1}H NMR spectrum (500 MHz, DMSO-d_6) of **3**

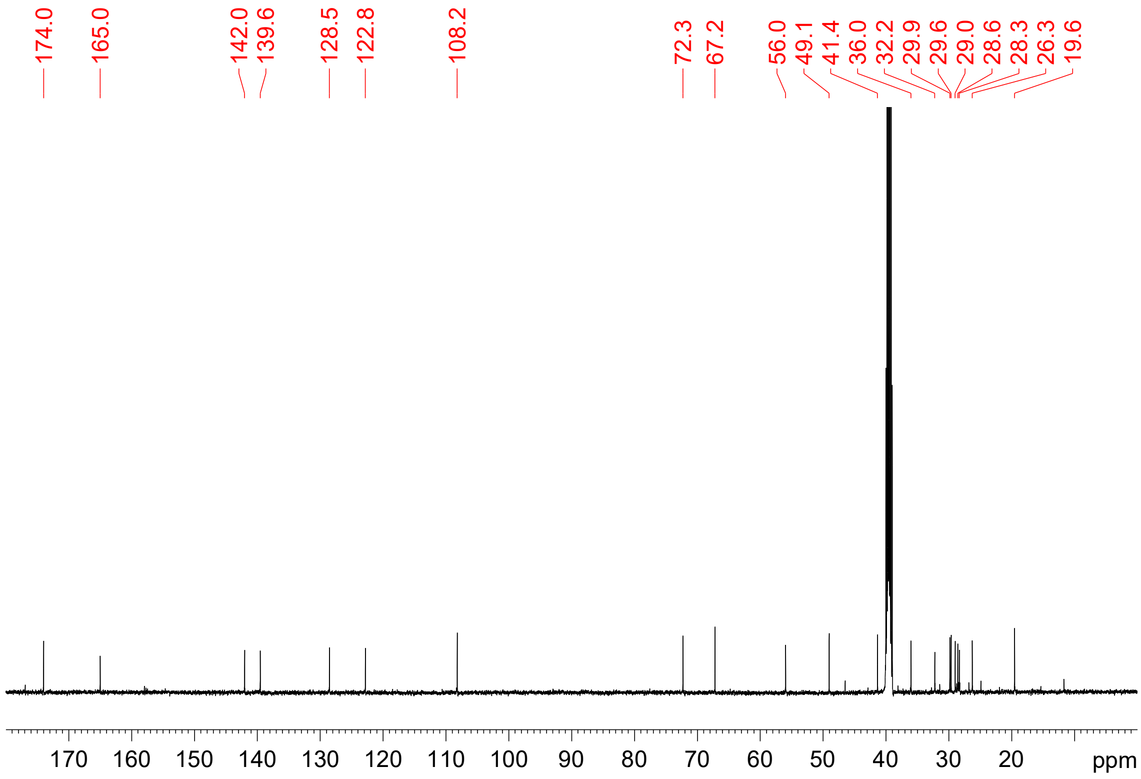

Figure S17. ^{13}C NMR spectrum (125 MHz, DMSO-d_6) of **3**

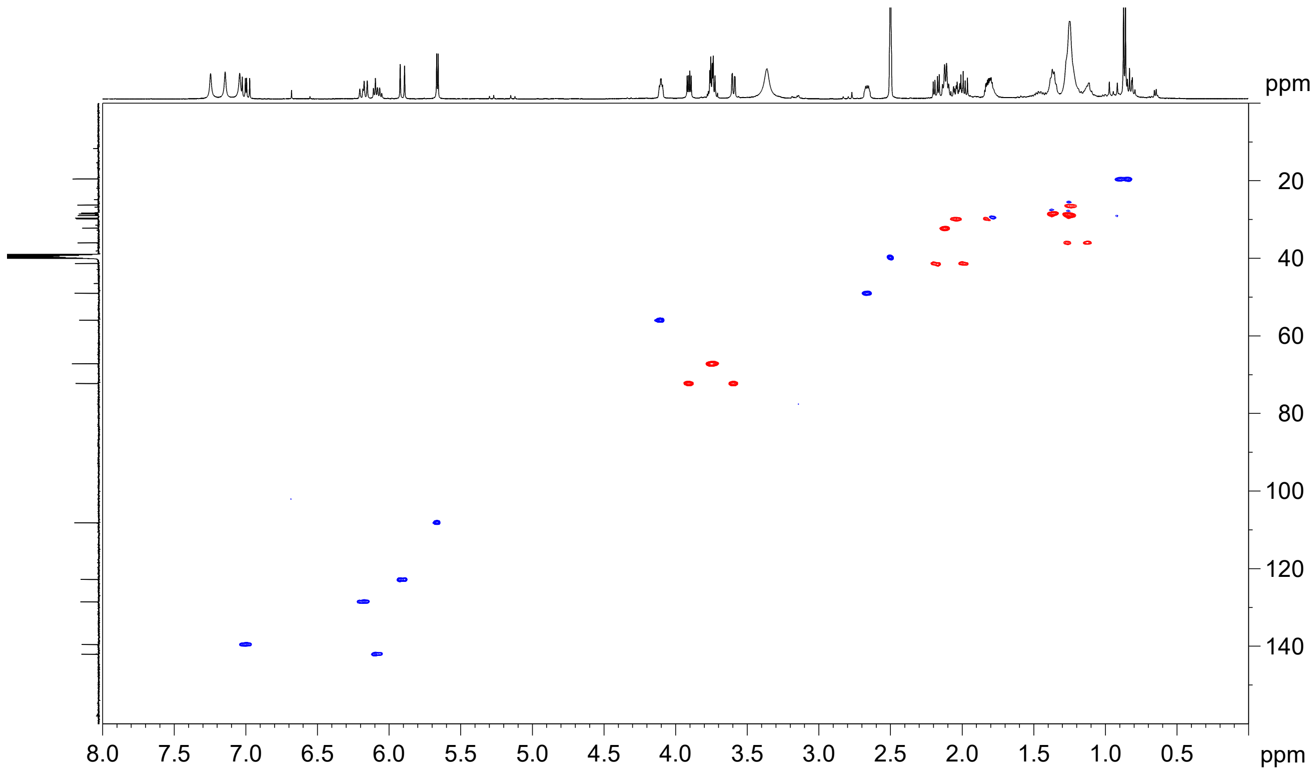

Figure S18. HSQC spectrum (500 MHz, DMSO-d_6) of **3**

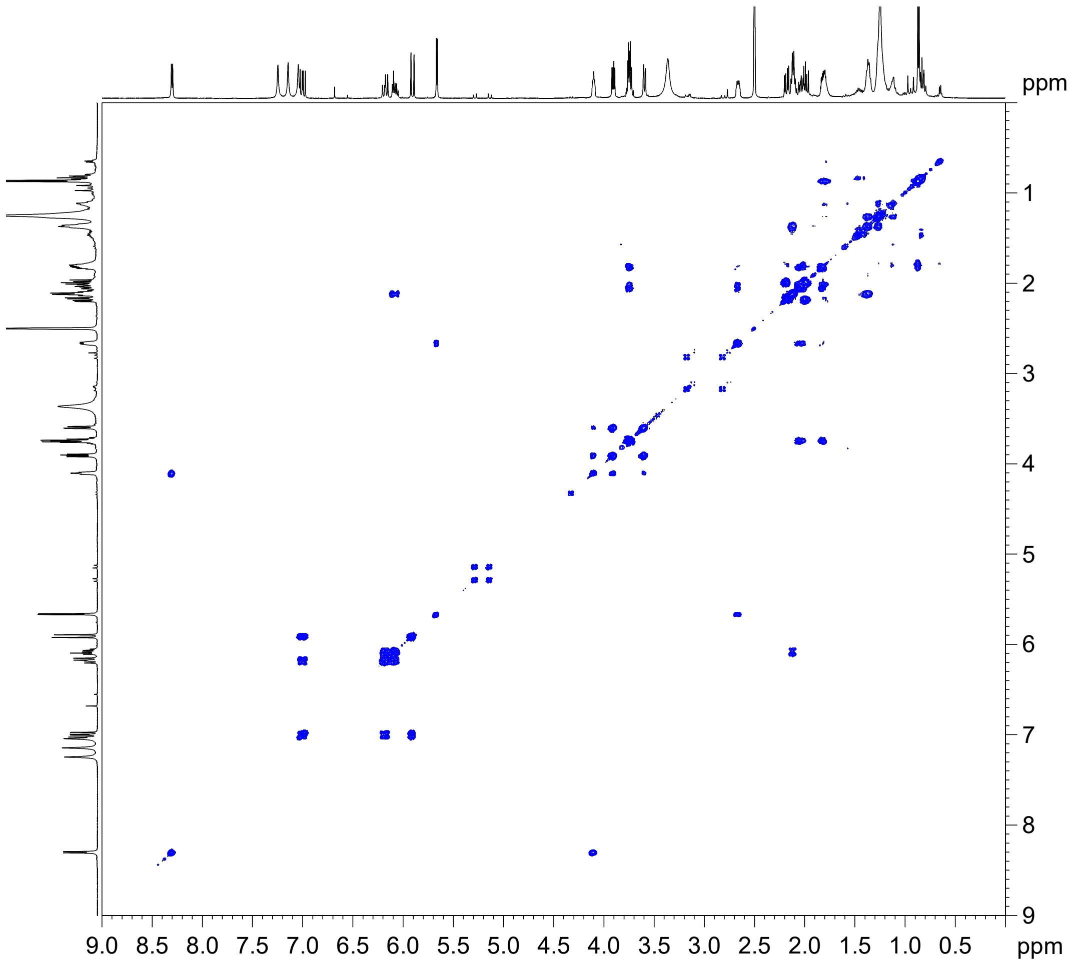

Figure S19. ^{1}H -^{1}H COSY spectrum (500 MHz, DMSO-d_6) of **3**

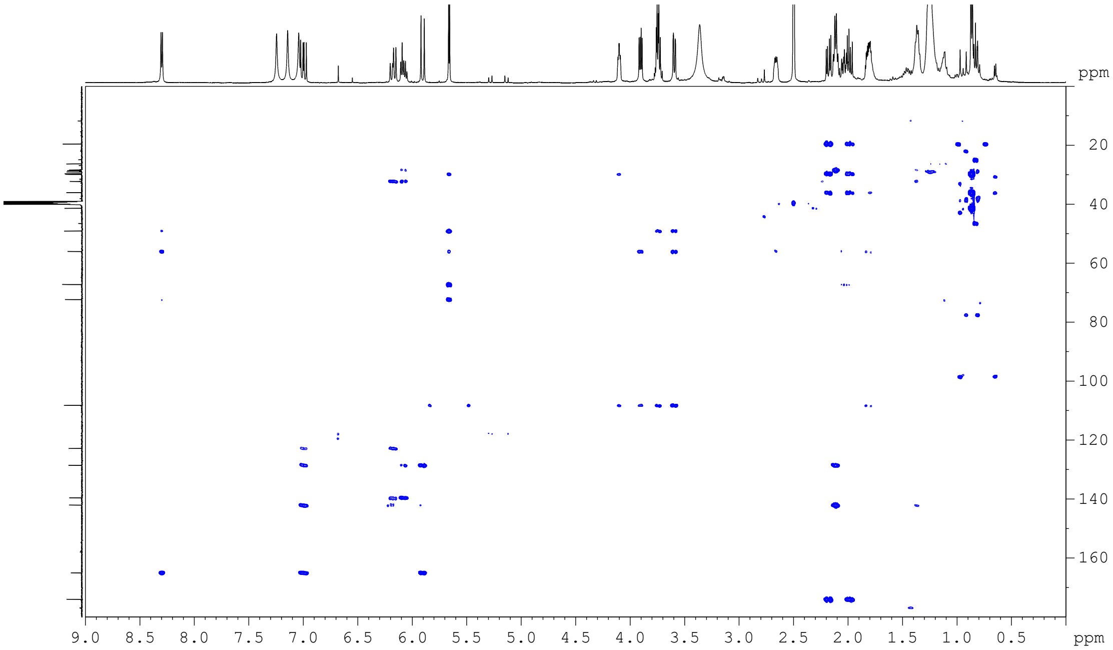

Figure S20. HMBC spectrum (500 MHz, DMSO-d_6) of **3**

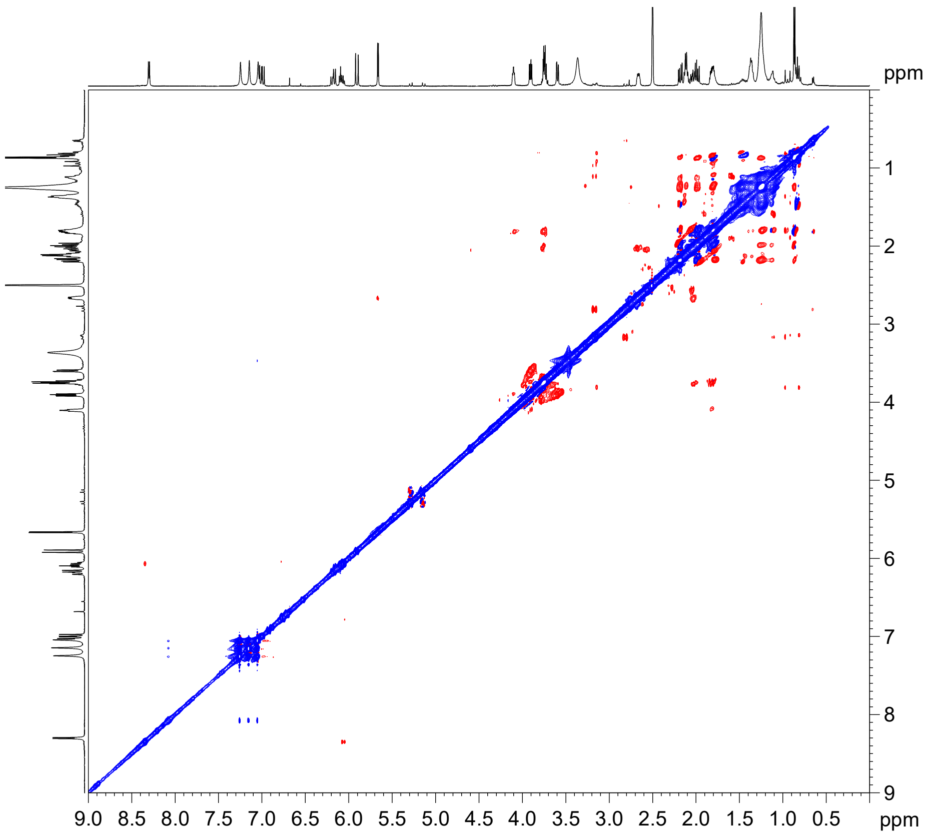

Figure S21. ROESY spectrum (500 MHz, DMSO-d_6) of **3**

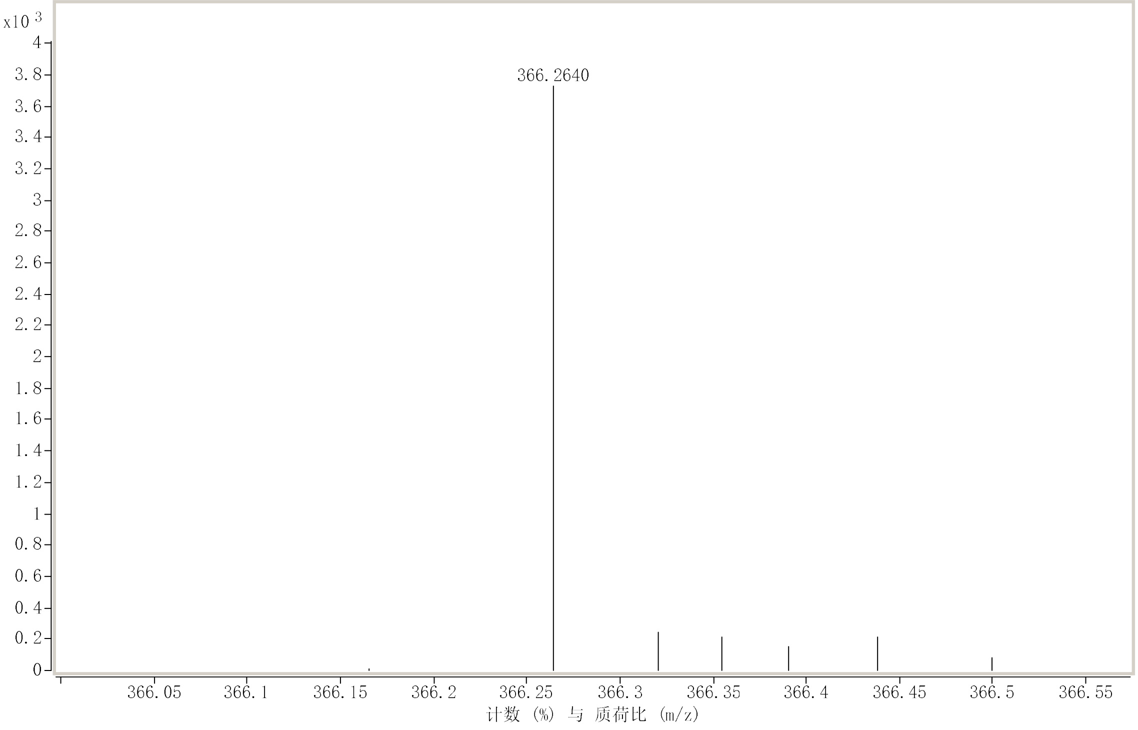

Figure S22. HRESIMS spectrum of **4**

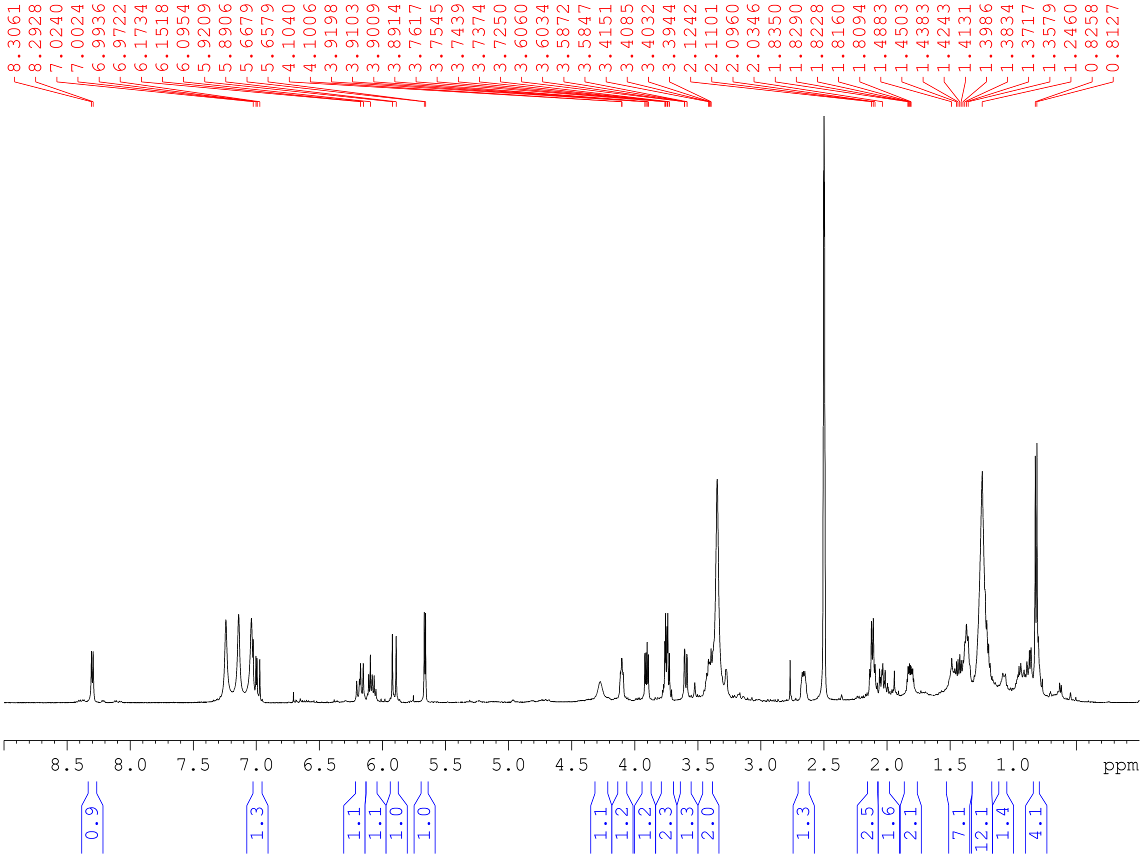

Figure S23. ^{1}H NMR spectrum (500 MHz, DMSO-d_6) of **4**

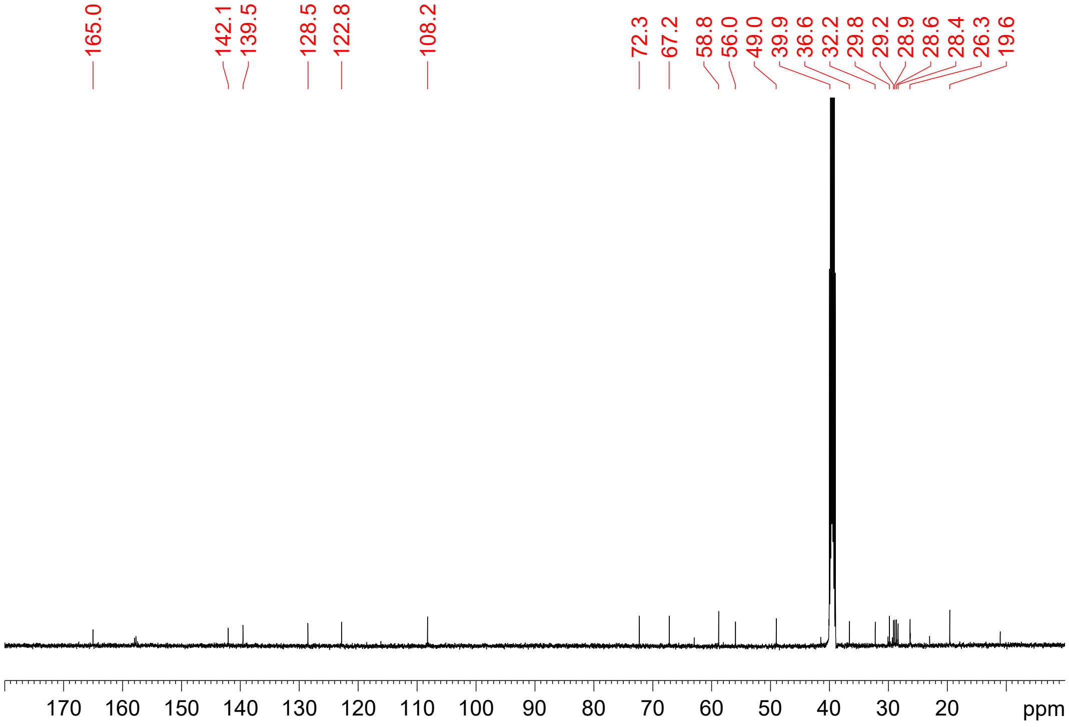

Figure S24. ^{13}C NMR spectrum (125 MHz, DMSO-d_6) of **4**

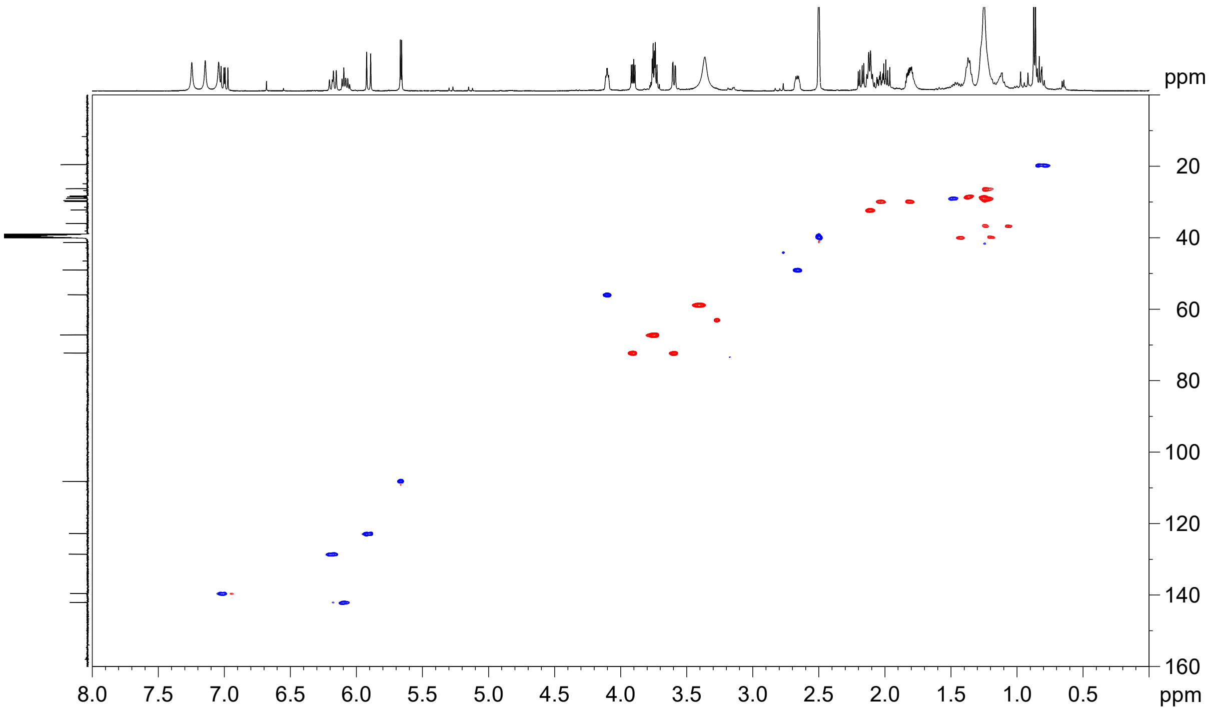

Figure S25. HSQC spectrum (500 MHz, DMSO-d_6) of **4**

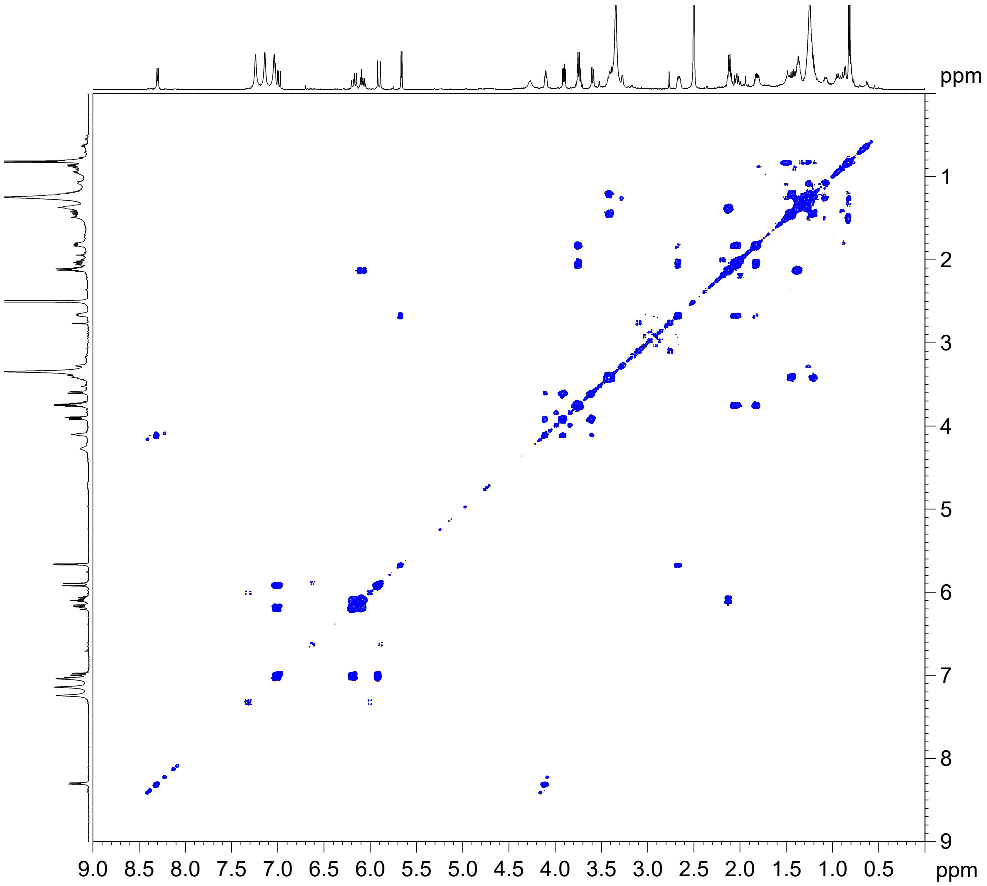

Figure S26. ^{1}H -^{1}H COSY spectrum (500 MHz, DMSO-d_6) of **4**

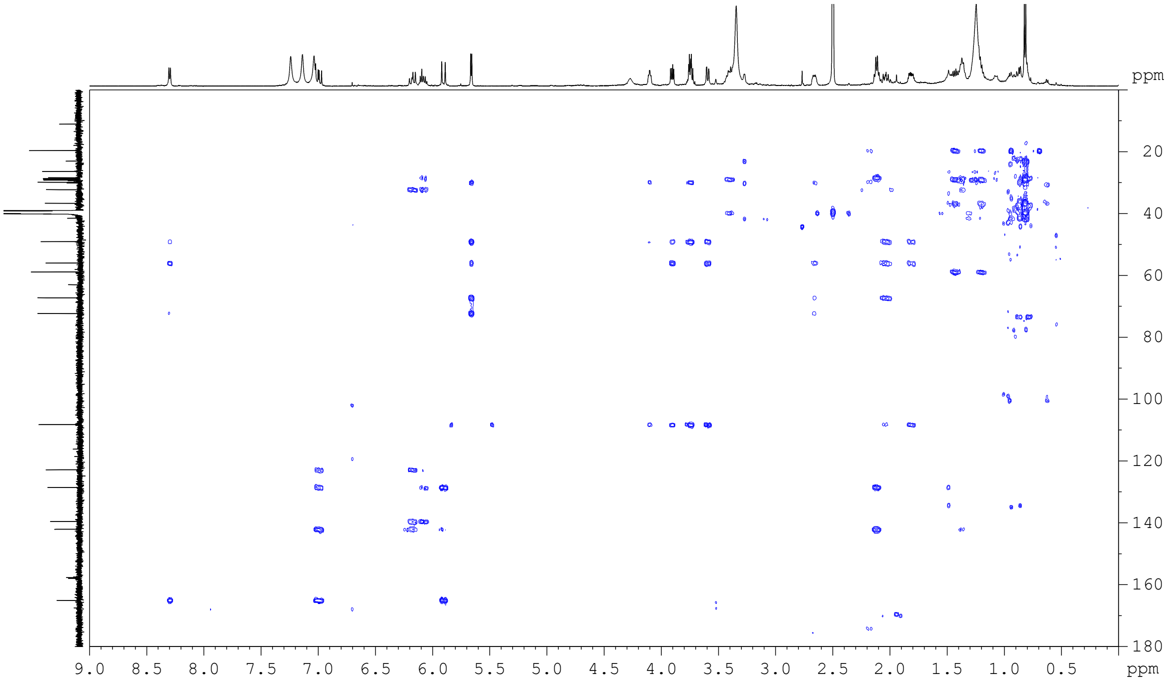

Figure S27. HMBC spectrum (500 MHz, DMSO-d_6) of **4**

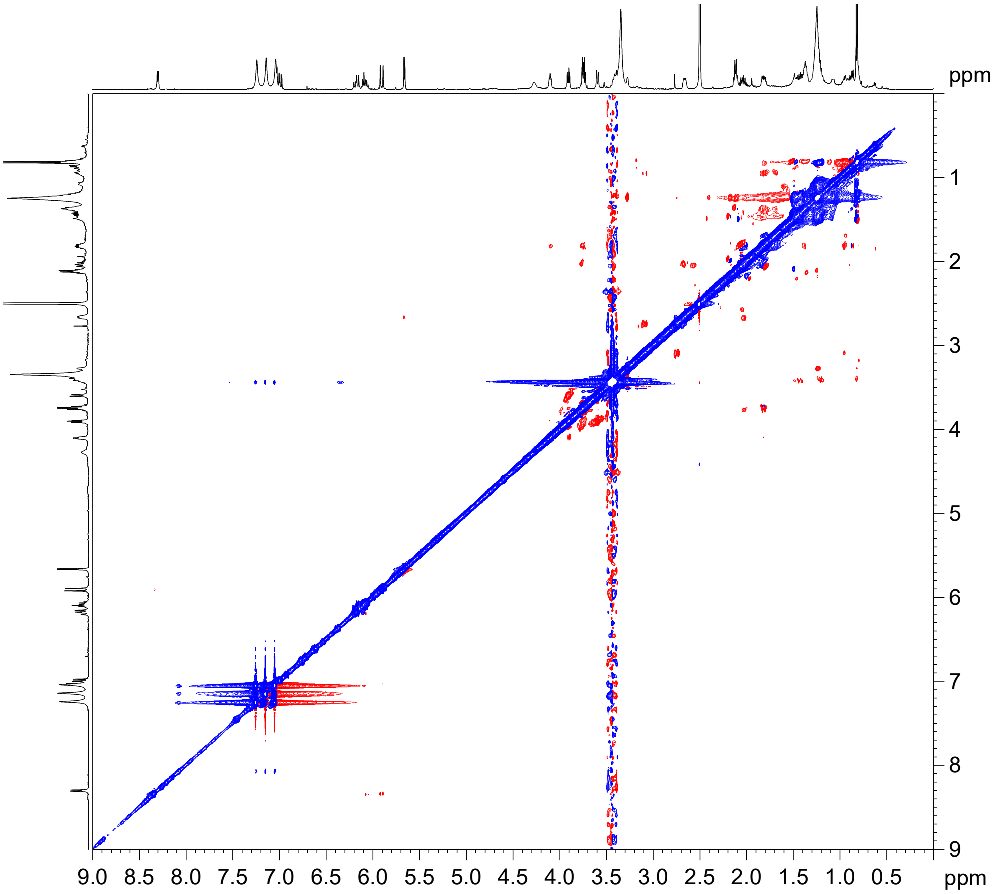

Figure S28. ROESY spectrum (500 MHz, DMSO-d_6) of **4**

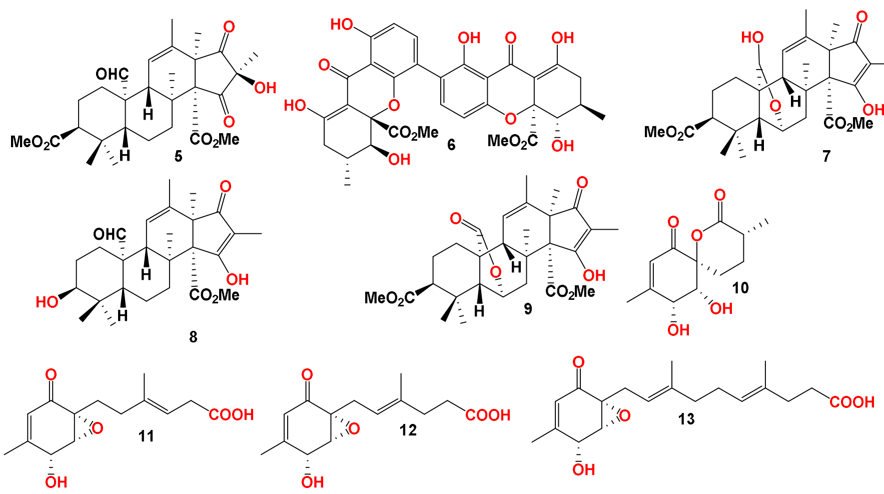

Figure S29. The structures of compounds 5-13.
